# ECM-integrin signalling instructs cellular position sensing to pattern the early mouse embryo

**DOI:** 10.1242/dev.200140

**Published:** 2022-01-13

**Authors:** Esther Jeong Yoon Kim, Lydia Sorokin, Takashi Hiiragi

**Affiliations:** 1 European Molecular Biology Laboratory (EMBL), Heidelberg 69117, Germany; 2 Collaboration for joint PhD degree between EMBL and Heidelberg University, Faculty of Biosciences, Universität Heidelberg, Heidelberg 69117, Germany; 3 Institute of Physiological Chemistry and Pathobiochemistry and Cells in Motion Interfaculty Centre (CiMIC), University of Muenster, Muenster 48149, Germany; 4 Institute for the Advanced Study of Human Biology (WPI-ASHBi), Kyoto University, Kyoto 606-8303, Japan

**Keywords:** Early mammalian development, Extracellular matrix, Cell fate specification, Patterning

## Abstract

Development entails patterned emergence of diverse cell types within the embryo. In mammals, cells positioned inside the embryo give rise to the inner cell mass (ICM), which eventually forms the embryo itself. Yet, the molecular basis of how these cells recognise their ‘inside’ position to instruct their fate is unknown. Here, we show that provision of extracellular matrix (ECM) to isolated embryonic cells induces ICM specification and alters the subsequent spatial arrangement between epiblast (EPI) and primitive endoderm (PrE) cells that emerge within the ICM. Notably, this effect is dependent on integrin β1 activity and involves apical-to-basal conversion of cell polarity. We demonstrate that ECM-integrin activity is sufficient for ‘inside’ positional signalling and is required for correct EPI/PrE patterning. Thus, our findings highlight the significance of ECM-integrin adhesion in enabling position sensing by cells to achieve tissue patterning.

## Abstract (precis)

**Summary:** The importance of patterned cell-extracellular matrix (ECM) interactions in early mouse development: ECM signals can modulate both cell fate and the relative spatial arrangement between cells.

## INTRODUCTION

Development results in an immense diversity of animal forms as fertilisation is followed by the organisation of cells into higher order structures. The emergence of complex patterns generally requires that cells continuously exchange signals with their surroundings to direct their fate and spatial orientation. Whereas transcriptional networks inform individual cell types, the position at which a cell lies is crucial for tissue patterning. Therefore, relays of spatial information are a ubiquitous requirement in developing systems, and a cell has to sense its position relative to its neighbours to support robust patterning. A cell may obtain positional information from a variety of sources, such as mechanochemical gradients, wave-like propagation of signalling activity, as well as direct adhesive interactions with its immediate environment (Jouve et al., 2002; Steinberg and Poole, 1981; Wolpert, 1969).

In particular, adhesive interactions with the extracellular matrix (ECM) are dynamically engaged during development and homeostatic turnover of tissues (Gattazzo et al., 2014; Walma and Yamada, 2020). The ECM consists of a network of various components, such as laminin, collagen IV and fibronectin, which serve to regulate cell behaviours ranging from migration, polarisation and survival to differentiation. Its significance is evident during development, in which loss of laminin chains, collagen IV or their respective receptors leads to early embryonic lethality in mice (Miner et al., 1998, 2004; Smyth et al., 1999; Williamson et al., 1997). Furthermore, laminin regulates the gene expression and spatial organisation of cells in several epithelial tissues (Klein et al., 1988; Streuli et al., 1995). In the gut, its loss leads to epithelial hyperplasia and an impaired stem cell pool, whereas provision of ECM through Matrigel supports the long-term culture of intestinal crypt organoids (Fields et al., 2019; Sato et al., 2009). Similarly, laminin is required for the correct positioning and maintenance of follicle stem cells in their niche within the *Drosophila* ovary (O'Reilly et al., 2008). As such, laminin as well as other ECM components have a conserved role in modulating the spatial organisation and behaviour of cells across diverse contexts.

The preimplantation mouse embryo is remarkable in its regulative capacity to preserve embryonic patterning against a drastic reduction in cell number (Solter and Knowles, 1975; Tarkowski, 1959; Tarkowski and Wróblewska, 1967). This implies dynamic readout of positional information by blastomeres to adjust their fate and spatial arrangement in response to perturbations. By the end of the preimplantation stage at embryonic day (E) 4.5, the embryo consists of an outermost trophectoderm (TE) monolayer enclosing a fluid-filled cavity and an inner cell mass (ICM). Within the ICM, the primitive endoderm (PrE) forms an epithelial monolayer lining the cavity, whereas epiblast (EPI) cells reside between the PrE and the overlying polar TE.

Cell position instructs the first lineage segregation in mouse development, when inner and outer cells become ICM and TE, respectively (Rossant and Tam, 2009; Tarkowski and Wróblewska, 1967). Prior to TE specification, the outer surface of the eight-cell embryo is marked by a polarised cortical domain enriched in phosphorylated ezrin, radixin and moesin (pERM), Par6 and atypical protein kinase C (aPKC) (Ducibella et al., 1977; Louvet et al., 1996; Vinot et al., 2005; Ziomek and Johnson, 1980). This apical domain is both necessary and sufficient for TE fate, and effectively serves as the ‘outside’ positional signal to prompt subsequent embryonic patterning (Alarcon, 2010; Korotkevich et al., 2017). However, insights into the specification of the ICM, which gives rise to the embryo itself, remain sparse thus far.

In contrast to apically polarised outer cells, inner cells are separated from the external environment and are instead enclosed by adhesive interactions with neighbouring cells. The earliest marker of ICM specification is the upregulation of *Sox2* within these inner cells of the embryo (Guo et al., 2010; Wicklow et al., 2014). Upon perturbation of internalisation or exposure to the external environment, early blastomeres default to a TE-like state (Korotkevich et al., 2017; Lorthongpanich et al., 2012; Stephenson et al., 2010; Tarkowski and Wróblewska, 1967), demonstrating that inside positioning of the blastomere is crucial for ICM specification.

## RESULTS

### Integrin and laminin chains are localised at the cell-cell interface

**Fig. 1.**
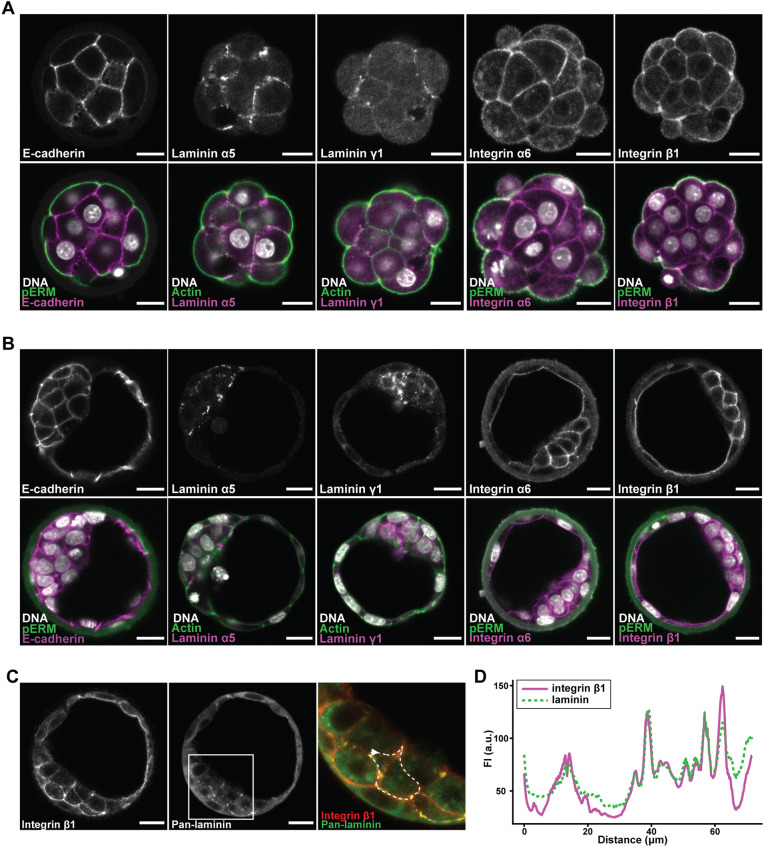
**Integrin and laminin are expressed in the morula and colocalise in the blastocyst.** (A,B) Localisation of E-cadherin, laminin chains α5 and γ1, and integrin α6 and β1 subunits in morulae (A) and blastocysts (B). pERM marks the cell-free apical surface of outer cells. (C) Co-immunostaining for integrin β1 and laminin (non-chain-specific) in the blastocyst marks their shared localisation at the cell-cell interface. Boxed outline indicates magnified region shown in right-hand image. (D) Representative intensity profile of integrin β1 and laminin around an inner cell (marked by dashed white line in C; arrowhead indicates starting point of measurement). Scale bars: 20 μm.

To study the ICM-inducing effects of the embryonic interior, we first examined proteins enriched at the cell-cell interface within the embryo. E-cadherin (also known as cadherin 1) was clearly localised to cell-cell contact sites from the morula to blastocyst stages, away from TE-associated apical domains enriched in pERM (Fig. 1A,B). Although E-cadherin is the major adhesive molecule that holds cells together irrespective of their fate (Filimonow et al., 2019; Larue et al., 1994; Shirayoshi et al., 1983; Stephenson et al., 2010), several studies have shown that ECM components are also present during this period of development (Dziadek and Timpl, 1985; Leivo et al., 1980; Morin and Sullivan, 1994; Sutherland et al., 1993). However, their significance is little understood.

We found that several laminin chains were enriched at the cell-cell interface in the morula and the ICM region of the blastocyst (Fig. 1A,B). Immunostaining indicated expression of laminin 511 in addition to the already-reported laminin 111, which are heterotrimers of constituent α5, β1, γ1 and α1, β1, γ1 chains, respectively (Cooper and MacQueen, 1983; Leivo et al., 1980; Miner et al., 2004; Smyth et al., 1999). Accordingly, subunits of the major laminin receptor, integrin α6β1, which binds both laminin 111 and 511, were similarly expressed in the preimplantation embryo (Fig. 1A,B) (Sutherland et al., 1993; Takizawa et al., 2017; Yamada and Sekiguchi, 2015). Close spatial association between laminin and integrin β1 fluorescence around inner cells identified ECM-integrin interactions as candidate ‘inside’ positional signals to blastomeres that could drive ICM specification (Fig. 1C,D).

### Exogenous ECM drives ICM specification and surface integrin α6β1 enrichment

**Fig. 2.**
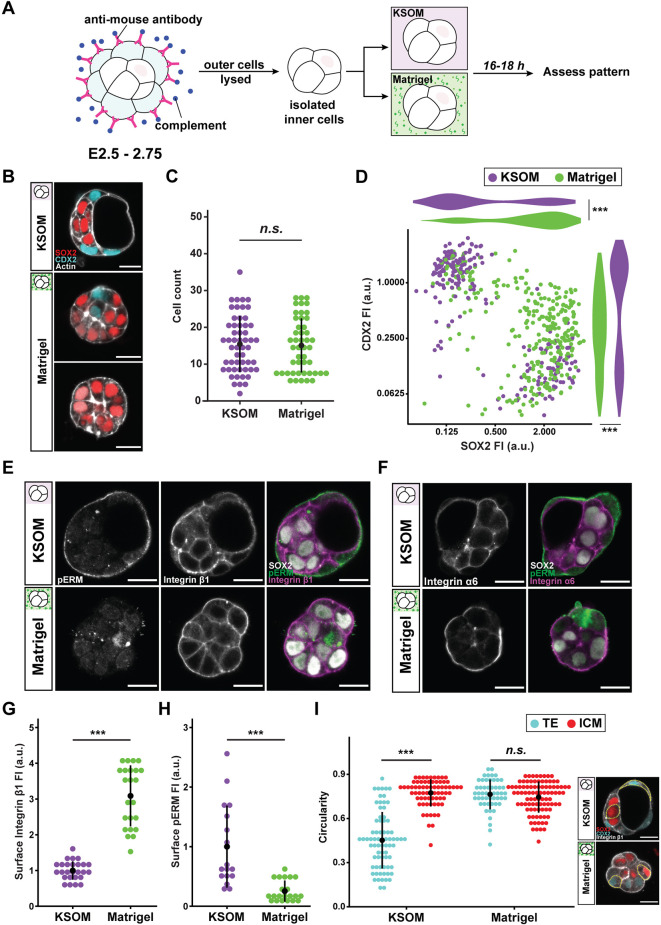
**Exogenous ECM drives ICM specification and surface integrin α6β1 enrichment.** (A) Schematic of experimental conditions and immunosurgery. Morula-stage embryos are recovered prior to ICM specification, and lysis of outer cells leaves behind isolated inner cells. Immediately following immunosurgery, inner cells are cultured in either standard KSOM mouse embryo media or Matrigel before assessment of patterning. (B) Representative images of TE-ICM fate specification following immunosurgery and culture in either control KSOM or Matrigel. CDX2 (cyan) marks TE fate, whereas SOX2 (red) marks ICM fate. In a few cases, Matrigel culture induces SOX2 upregulation across the entire cell cluster (bottom panel). (C) Total cell count after immunosurgery and culture in either KSOM (purple) or Matrigel (green). Each data point represents the cell number of the inner cell cluster cultured from a single embryo; *n*=101 embryos. (D) Scatterplot and adjacent violin plots show normalised fluorescence intensities of CDX2 and SOX2 measured for each cell cultured in either KSOM (purple) or Matrigel (green). Data analysed with Mann–Whitney *U*-test; ***P<0.001; *n*=43 embryos (*n*=912 cells). (E,F) Representative images of pERM (apical marker), integrin β1 and integrin α6 localisation in cultured inner cells. (G,H) Quantification of surface enrichment of integrin β1 and pERM based on fluorescence intensity [*n*=49 (F) and *n*=38 (G) embryos]. (I) Circularity as a descriptor of cell shape measured for individual TE- and ICM-specified cells across the two culture conditions; *n*=46 embryos (*n*=288 cells). Data in C,G-I analyzed with two-sided Student's *t*-test; n.s., non-significant; ***P<0.001; error bars show mean±s.d. in each case. Scale bars: 20 μm. See also Fig. S1.

To test whether the ECM can present ‘inside’ positional signals to drive ICM specification, we sought to mimic the inner environment of the embryo by providing ECM to cells through Matrigel, which is rich in laminin 111 (Orkin et al., 1977; Timpl et al., 1979). Embryos were recovered at the morula stage prior to marked upregulation of *Sox2* in inner cells, and TE-specified outer cells were removed by immunosurgery (Fig. 2A). Immunosurgery not only isolates naïve inner cells, but also alters their positional identity by exposing them to the external environment (Solter and Knowles, 1975). Subsequent culture of these cells in standard potassium simplex optimized medium (KSOM; LifeGlobal, LGGG-050) fully restored inside-outside patterning. CDX2-positive TE cells surrounded SOX2-positive ICM cells and often a small fluid-filled cavity, reminiscent of blastocysts (Fig. 2B, top panel). In this way, these isolated cells displayed robust regulative capacity by restoring embryonic patterns seen in whole counterparts.

In contrast, however, the TE layer was not restored in the presence of Matrigel. Instead, isolated cells formed a compact mass in which most nuclei were SOX2 positive, irrespective of cell position (Fig. 2B). CDX2-positive cells were fewer and clustered at the periphery, whereas fluid-filled cavities were noticeably absent. Moreover, samples entirely composed of SOX2-positive cells were also observed across independent experiments, albeit at low frequency (nine out of 97; 9.3%) (Fig. 2B, bottom panel). Total cell numbers were comparable between the two conditions (Fig. 2C), indicating that Matrigel does not have adverse effects on cell survival or proliferation.

Besides expression of *Cdx2* and *Sox2*, TE and ICM cells are distinguishable by differential Hippo signalling (Nishioka et al., 2009; Wicklow et al., 2014). In inner cells, Hippo signalling resulted in phosphorylation and cytoplasmic retention of YAP1. In outer cells, Hippo signalling was inactive, and YAP1 translocated to the nucleus to drive downstream transcription of *Cdx2*. Consistent with increased *Sox2* expression, nuclear YAP1 localisation was diminished in Matrigel culture (Fig. S1A). Furthermore, quantitative analysis of individual nuclei for levels of each fate marker confirmed the significant increase in *Sox2* expression in Matrigel (Fig. 2D). These findings demonstrate that exogenously supplied ECM provides ‘inside’ positional cues sufficient to drive ICM specification following immunosurgery even in cells that are physically positioned ‘outside’.

Earlier studies noted that TE specification is preceded by ready polarisation of the outer surface after perturbations such as immunosurgery (Stephenson et al., 2010; Wigger et al., 2017). In agreement with this, pERM was enriched on the outer surface of isolated cells cultured in KSOM, whereas integrin β1 was limited to cell-cell interfaces (Fig. 2E, top panels). Distinct and mutually exclusive localisation of pERM and integrin β1 is consistent with the apicobasal polarity that accompanies inside-outside patterning in the whole embryo. In contrast, however, Matrigel led to significant enrichment of integrin β1 on the outer surface, whereas peripheral pERM was significantly diminished [Fig. 2E (bottom panels) and Fig. 2G,H]. Discontinuous patches of pERM were sometimes present on the surface, which generally coincided with CDX2-positive or SOX2-negative nuclei (Fig. S1B). Integrin α6 localisation was comparable to that of integrin β1 (Fig. 2F), whereas E-cadherin was limited to cell-cell interfaces regardless of culture conditions (Fig. S1C). These results suggest that Matrigel, particularly its constituent laminin, brings its receptor integrin α6β1 to the surface in lieu of apical polarity proteins, to the benefit of ‘inside’ cells.

Whereas ICM cells are approximately isotropic in shape, TE cells are generally oblong under control conditions because they are stretched around the ICM or the fluid-filled cavity (Chan et al., 2019; Niwayama et al., 2019). However, Matrigel abrogated this difference in circularity between TE and ICM cells. The presence of round TE cells in Matrigel culture suggests that fate specification in this setting is not dependent on cell shape (Fig. 2I).

### Integrin β1 activity is required for ECM-induced ICM specification

**Fig. 3.**
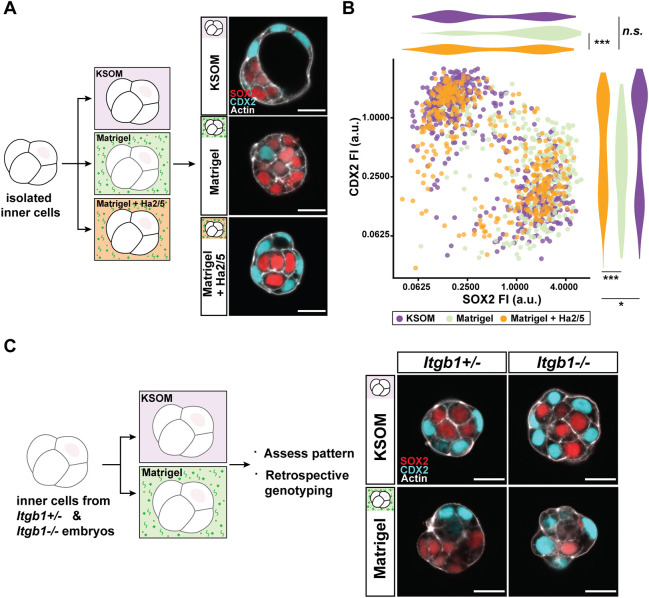
**ICM induction by Matrigel is dependent on integrin β1 activity.** (A) Schematic of experimental conditions and representative images of TE-ICM patterning upon administration of an integrin β1 function-blocking antibody, Ha2/5 (10 µg/ml), with Matrigel. (B) Scatterplot and adjacent violin plots show normalised fluorescence intensities of CDX2 and SOX2 measured for each cell cultured in either KSOM (purple), Matrigel only (light green) or Matrigel with Ha2/5 (orange). Plot represents data combined from *n*=93 embryos (*n*=1332 cells). Data for KSOM and Matrigel are duplicated from Fig. 2D for ease of comparison. Data analysed with Mann–Whitney *U*-test; n.s., non-significant; *P<0.05, ***P<0.001. (C) Schematic of experimental conditions and representative images of inner cells isolated from *Itgb1* transgenic embryos cultured in either KSOM or Matrigel. Each sample is genotyped retrospectively to identify *Itgb1^−/−^* samples. *Itgb1^+/−^* samples serve as littermate controls; *n*=25 embryos (14 *Itgb1^+/−^* and 11 *Itgb1^−/−^*). Scale bars: 20 μm. See also Fig. S2.

To test whether the activity of surface-enriched integrin α6β1 is required for ICM induction by Matrigel, integrin β1 was inhibited with the function-blocking antibody, Ha2/5 (Mendrick and Kelly, 1993). Administration of Ha2/5 almost completely attenuated the aforementioned effects of Matrigel. Outer cells polarised and became TE specified, whereas ICM specification was confined to inner cells (Fig. 3A,B; Fig. S2A). Thus, cells cultured in Matrigel with Ha2/5 were indistinguishable from control samples. Similar observations were made upon inhibition of integrin α6 and assessment of YAP1 localisation (Fig. S2B,C) (Sonnenberg et al., 1987), in which a continuous outer TE layer was restored despite the presence of Matrigel.

Furthermore, upon genetic ablation of *Itgb1*, integrin β1-deficient cells were refractory to the effects of Matrigel (Raghavan et al., 2000). Unlike cells isolated from *Itgb1^+/−^* littermate controls, which exhibited increased ICM specification in Matrigel, inside-outside patterning was restored among *Itgb1^−/−^* cells (Fig. 3C). These results indicate that ‘inside’ positional signals provided by the ECM require recognition through integrin α6β1 activity to drive ICM specification.

### Integrin β1 activity is not required for initial specification of ICM but is for EPI-PrE patterning *in vivo*

**Fig. 4.**
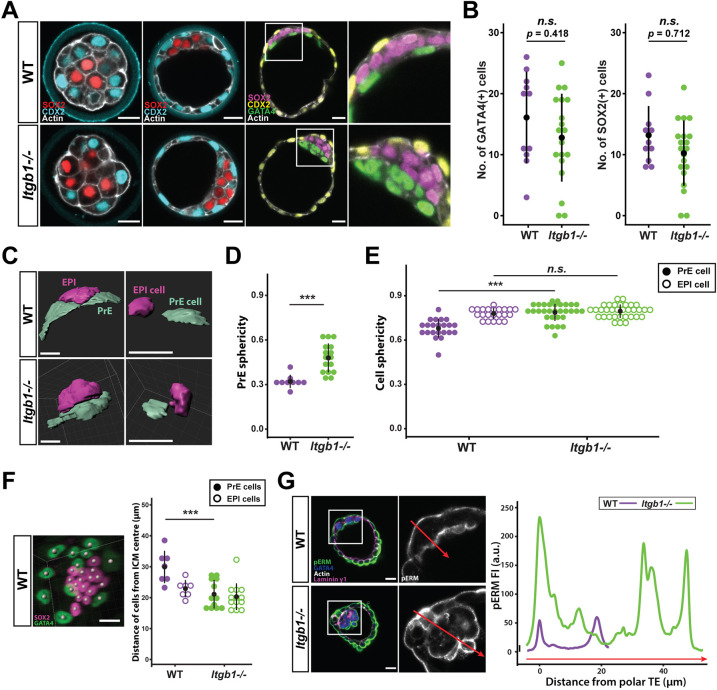
**EPI-PrE patterning in the late blastocyst *in vivo* requires integrin β1.** (A) Representative images of lineage patterning in preimplantation-stage WT and *Itgb1^−/−^* embryos through morula, early and late blastocyst stages. Boxed outline indicates region magnified in right-hand images. (B) Cell count of GATA4-expressing PrE cells and SOX2-expressing EPI cells within the ICM of WT and *Itgb1^−/−^* late blastocysts; *n*=31 embryos (11 WT, 20 *Itgb1^−/−^*). (C) Representative images of segmented PrE (cyan) and EPI (magenta), as well as individual segmented cells in E4.0 blastocysts on Imaris. (D,E) Sphericity of PrE tissue and individual cells of the ICM, acquired from segmented surfaces, compared across WT and *Itgb1^−/−^* blastocysts at E4.0; *n*=25 embryos in D and *n*=108 cells from 17 embryos in E. (F) Representative image of EPI(SOX2) and PrE(GATA4) nuclei detected in 3D on Imaris. Individual *x*-, *y*-, and *z*-coordinates of detected spots are used to calculate the distance of each nucleus from the centre of the ICM. On the plot, each dot represents the average distance value from all PrE or EPI cells from one embryo; *n*=25 embryos. (G) Representative image of morphology and apical polarity of the ICM in WT and *Itgb1^−/−^* blastocysts at E4.0. Accompanying intensity profile shows the distribution of pERM across the ICM along the line of interest (indicated by red arrows). Data in B,D-F analyzed with two-sided Student's *t*-test; n.s., non-significant; ***P<0.001; error bars show mean±s.d. in B,D,E and mean±s.e.m. in F. Scale bars: 20 µm. See also Fig. S3.

Earlier observation of integrin β1-mutant mice showed embryonic lethality post-implantation but apparently normal development through the preimplantation stage (Fässler and Meyer, 1995). Accordingly, we found TE-ICM patterning and overall morphology to be comparable between wild-type (WT) and *Itgb1*^−/−^ embryos during most of the preimplantation stage (Fig. 4A). Examination of morula-stage embryos specifically for the presence of the active conformation of integrin β1 revealed that integrin was mainly active on the basal side of outer cells (Fig. S3A) (Bazzoni et al., 1995; Humphries et al., 2005). These data consistently suggest that, although an abundance of ECM signals is sufficient to drive increased ICM specification, as shown earlier, it is not strictly required for TE/ICM patterning *in vivo*.

However, defects were observed upon close examination of mutant blastocysts towards the end of the preimplantation stage, around E4.0. Within the mature ICM of a WT blastocyst, PrE cells formed an epithelial monolayer that was apically polarised towards the blastocyst cavity, whereas EPI cells were sheltered between the PrE and the overlying polar TE (Fig. 4A). The respective numbers of EPI and PrE cells were not significantly affected by integrin β1 deficiency on average (Fig. 4B). On rare occasions, we observed *Itgb1^−/−^* blastocysts with severe disruption of the ICM, in which cell numbers were drastically reduced or EPI/PrE ratios were skewed (Fig. S3B). However, the most consistent mutant phenotype was the failure of PrE cells to resolve into a monolayer epithelium (Fig. 4A).

For further characterisation of altered ICM morphology, we segmented EPI and PrE tissues as well as individual cells by using fluorescence signals from membrane and lineage markers (Fig. 4C). Instead of flattening out beneath the EPI and TE, *Itgb1^−/−^* PrE tissues and individual PrE cells were less spread and more spherical in shape (Fig. 4C-E). Segmented EPI tissues were also more rounded in mutants (Fig. S5A). Furthermore, detection of ICM nuclei indicated that *Itgb1^−/−^* PrE nuclei were more closely clustered around the centre of the ICM compared with WT (Fig. 4F). These observations indicate that integrin β1 deficiency results in rounded ICM morphology, stemming from multi-layered PrE tissues as well as shape changes at the level of individual PrE cells.

In addition, the failure to form a spread PrE monolayer was accompanied by disrupted polarity in *Itgb1^−/−^* embryos. Whereas apical PKCζ intensity peaked at the PrE surface facing the blastocyst cavity, its distribution was broader across the mutant PrE layer compared with WT (Fig. S3C) (Saiz et al., 2013). In contrast, PKCζ distribution in the TE was comparable between genotypes (Fig. S3D). Similar observations were made of pERM localisation. Whereas WT embryos exhibited a bimodal pERM distribution, in which fluorescence intensity peaked at the apical surface of the polar TE and PrE, *Itgb1^−/−^* profiles exhibited multiple peaks (Fig. 4G). Therefore, although integrin β1 is not required for initial specification of the ICM *in vivo*, it is required for subsequent patterning among EPI and PrE cells inside the blastocyst. In particular, it is required for the organised formation of a polarised epithelial PrE monolayer. These findings reveal that defects that underlie the reported post-implantation lethality of *Itgb1^−/−^* embryos in fact arise prior to implantation.

### Exogenous ECM leads to EPI cells dwelling on the surface of the ICM

In contrast to TE-ICM specification, subsequent EPI-PrE specification within the ICM is not cell position dependent because respective cells emerge in a salt-and-pepper pattern (Chazaud et al., 2006; Plusa et al., 2008). Nevertheless, positional information remains pertinent because EPI and PrE cells must resolve into a distinct spatial pattern, as described above. Given the requirement for integrin β1 during this latter process, as demonstrated by mutant blastocysts, we tested whether EPI and PrE cells are also receptive to exogenous ECM as a positional cue.

**Fig. 5.**
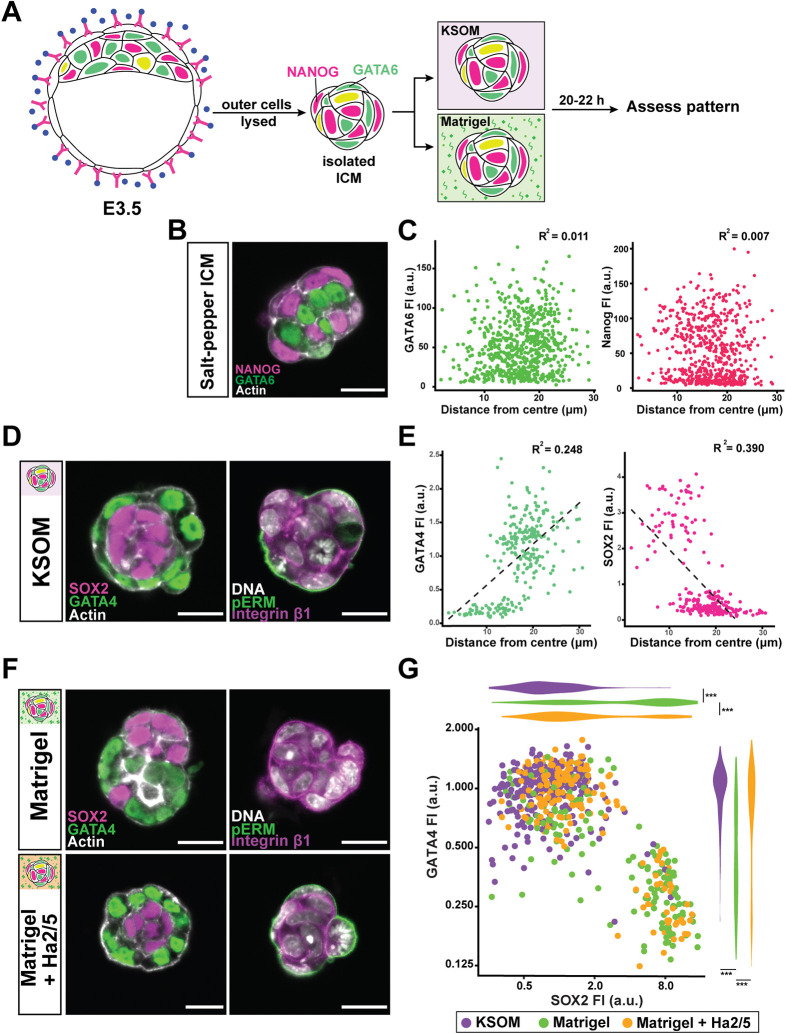
**EPI-PrE patterning is sensitive to Matrigel and integrin β1 activity.** (A) Schematic of experimental conditions with immunosurgery of blastocysts, where pink is NANOG, green is GATA6, and yellow indicates their co-expression. Blastocysts are subjected to immunosurgery to isolate salt-and-pepper-stage ICMs. Isolated ICMs are cultured in either KSOM or Matrigel before assessment of patterning. (B) Representative image of an isolated salt-and-pepper ICM expressing the early EPI marker NANOG (magenta) and early PrE marker GATA6 (green). (C) Scatterplots showing fluorescence intensities of GATA6 and NANOG in relation to cell position within an isolated salt-and-pepper ICM. Position is measured as the distance between each nucleus and the centre of the ICM. ICMs from *n*=28 embryos (*n*=658 cells). (D) Representative images of EPI-PrE arrangement and apicobasal polarity of ICMs following culture in KSOM. SOX2 (magenta) marks EPI cells, and GATA4 (green) marks PrE cells on the left panel. (E) Scatterplots showing fluorescence intensities of GATA4 and SOX2 in relation to cell position following culture of ICMs in KSOM; ICMs from *n*=35 embryos (*n*=765 cells). (F) Representative images of EPI-PrE spatial arrangement and apicobasal polarity of ICMs following culture in Matrigel or Matrigel with the integrin β1 function-blocking antibody, Ha2/5 (10 µg/ml). (G) Scatterplot and adjacent violin plots show normalised fluorescence intensities of GATA4 and SOX2 measured for each peripherally located cell cultured in KSOM (purple), Matrigel (green) or Matrigel with Ha2/5 (orange). Plot represents data from ICMs from *n*=59 embryos (*n*=1664 cells), analysed with Mann–Whitney *U*-test; ***P<0.001. Data in C,E analysed with Pearson's correlation coefficient; *P*<0.0001 for both GATA4 and SOX2 in E. Scale bars: 20 μm. See also Fig. S4.

The transcription factors NANOG and GATA6 are early markers of EPI and PrE fate, respectively. When ICMs were isolated from blastocysts at E3.5, NANOG- and GATA6-positive nuclei, as well as double-positive nuclei, were intermixed (Fig. 5A,B). The distance between each nucleus from the centre of the ICM showed no correlation with expression level of cell fate markers (Fig. 5C), as expected from a salt-and-pepper pattern.

Following immunosurgery and culture, the salt-and-pepper distribution of fates resolved into a pattern in which polarised GATA4-positive PrE surrounded the SOX2-positive EPI (Fig. 5D). Positional distinction between EPI and PrE was evident based on cell fate marker expression relative to distance from the ICM centre (Fig. 5E). Given the small size of the ICM, correlation coefficient values appeared low, but there was significant positive correlation between GATA4 expression and nuclear distance from the ICM centre, whereas a negative correlation was observed for SOX2 expression. In contrast, Matrigel markedly disrupted this spatial arrangement (Fig. 5F, top-left panel). EPI cells were no longer confined to the interior, but frequently found at the surface. In Matrigel, spatial position could not distinguish the two lineages because PrE cells tend to be closer and EPI cells further from the centre of the cultured ICM compared with their respective counterparts in KSOM (Fig. S4A). In addition, quantitative analysis of fate in peripherally located cells indicated that, whereas most outer cells were GATA4 positive in control conditions, a significant portion expressed SOX2 in Matrigel culture (Fig. 5G). Furthermore, apical polarity of the ICM surface was replaced by integrin β1 enrichment (Fig. 5F, top-right panel), as observed from culture of inner cells at the earlier stage.

As with TE-ICM patterning, the effects of Matrigel on EPI-PrE patterning were dependent on integrin β1 activity. Administration of Ha2/5 restored a peripheral polarised PrE layer in the presence of Matrigel [Fig. 5F (bottom panels) and Fig. 5G], as did genetic ablation of *Itgb1* (Fig. S4B). These observations demonstrate that ECM-integrin adhesion provides crucial positional signals to regulate EPI-PrE patterning within the ICM, consistent with its role in ICM-TE patterning following immunosurgery, and as seen in whole *Itgb1^−/−^* blastocysts.

### Integrin and laminin signal together in the preimplantation embryo

Given our findings, we next sought to identify the extracellular protein component involved in ECM and integrin-mediated position sensing *in vivo*.

**Fig. 6.**
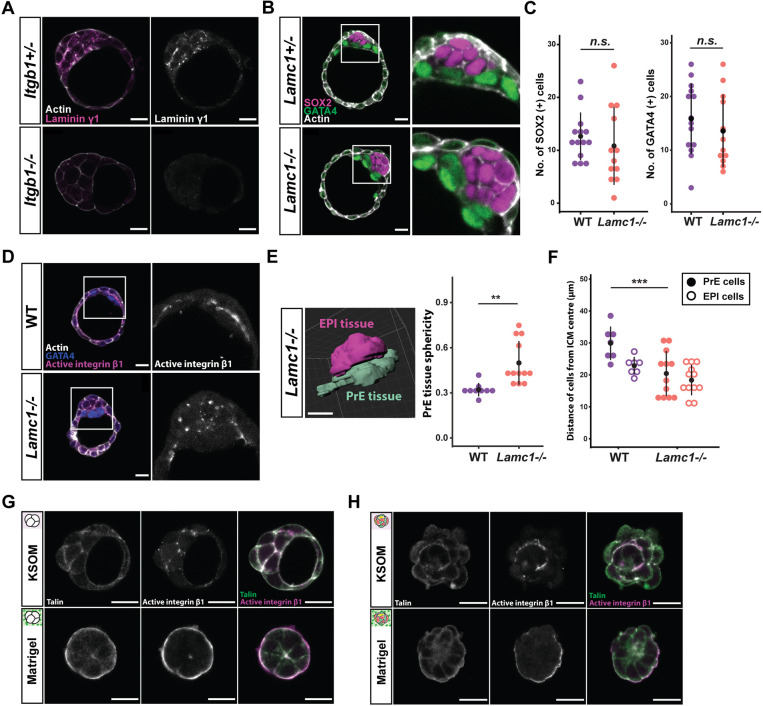
**Integrin and laminin signal together in the preimplantation embryo.** (A) Representative images of laminin γ1 chain localisation in *Itgb1^+/−^* or *Itgb1^−/−^* blastocysts at E4.0. *Itgb1^+/−^* embryos serve as littermate controls. (B) Representative images of EPI-PrE patterning within the ICM of WT and *Lamc1*^−/−^ blastocysts at E4.0. *Lamc1^+/−^* embryos serve as littermate controls. (C) Cell count of GATA4-expressing PrE cells and SOX2-expressing EPI cells within the ICM of WT and *Lamc1^−/−^* blastocysts; *n*=26 embryos (14 WT, 12 *Lamc1^−/−^*). (D) Representative images of the distribution of active integrin β1 (9EG7 antibody) within the ICM of WT and *Lamc1^−/−^* blastocysts at E4.0. (E) Representative image of segmented PrE and EPI tissues in a *Lamc1^−/−^* blastocyst at E4.0 on Imaris. Plot displays the sphericity of PrE tissue, calculated from segmented surfaces; *n*=22 embryos. (F) Dot plot shows distance of PrE and EPI nuclei from the centre of the ICM. Each dot represents the average distance value from all PrE or EPI cells from one embryo; *n*=38 embryos. (G-H) Representative images of localisation of talin and the active conformation of integrin β1 (9EG7 antibody), following immunosurgery at either E2.5 (G) or E3.5 (H) and culture in KSOM or Matrigel. Data in C,E,F analysed with two-sided Student's *t*-test; n.s., non-significant; **P<0.01, ***P<0.001. Error bars show mean±s.d. in C,E and mean per embryo±s.e.m. in F. Scale bars: 20 μm.

Although laminin itself is a ligand for integrin, integrin β1 is required for the deposition of heterotrimeric laminin into the intercellular space, which, in turn, can bring its cell surface receptors together (Aumailley et al., 2000; Li et al., 2002). Accordingly, intercellular laminin, as judged by strand-like laminin γ1 signal in the ICM, was diminished in *Itgb1^−/−^* embryos (Fig. 6A). Given that the requirement for laminin γ1, encoded by *Lamc1*, is shared by both laminin isoforms (laminin 111 and laminin 511) assembled during the preimplantation stage, our model predicted integrin signalling to be impaired in *Lamc1^−/−^* embryos.

Examination of *Lamc1^−/−^* blastocysts revealed that PrE cells failed to resolve into an epithelial monolayer (Fig. 6B), despite the number of PrE and EPI cells being comparable to WT (Fig. 6C). As predicted, *Lamc1^−/−^* mutants exhibited diminished integrin β1 activity on the basal side of the PrE (Fig. 6D). Linear distribution of active integrin β1 was pronounced at the EPI-PrE boundary in WT ICM. In contrast, however, the signal was punctate and often weak within *Lamc1^−/−^* ICM.

Furthermore, similar to their *Itgb1^−/−^* counterparts, segmented PrE and EPI tissues were more spherical in *Lamc1^−/−^* mutants (Fig. 6E; Fig. S5A), and individual cells were also more rounded compared with WT cells (Fig. S5B). PrE cells were more closely clustered around the centre of the ICM (Fig. 6F), and such failure to form a spread PrE monolayer was accompanied by disrupted apicobasal polarity, as seen in *Itgb1^−/−^* mutants (Fig. S5C,D). Thus, the close resemblance between *Itgb1^−/−^* and *Lamc1^−/−^* blastocysts supports a model in which intercellular laminin provides crucial positional signals that are interpreted by cells via integrin activity to instruct patterning of the ICM.

The cytoplasmic domain of integrins interacts with myriad proteins. Among these, talin plays a key role in linking integrin to the cytoskeleton, and recruits other integrin-associated proteins, such as vinculin, for signalling (Calderwood et al., 1999; Humphries et al., 2007). Indeed, where the active conformation of integrin β1 is enriched on the surface by Matrigel culture, the talin signal is also increased, both during ICM induction and in surface-positioned EPI cells (Fig. 6G and H). Together, these results suggest that talin may be one of the components involved in relaying positional information within the early embryo to affect patterning.

## DISCUSSION

During mouse preimplantation development, ICM-TE specification follows an inside-outside pattern, whereas EPI and PrE cells initially emerge in an intermixed manner before becoming spatially segregated. Despite this difference, however, we show that cells maintain sensitivity to ECM-integrin signals throughout the preimplantation period to gain positional information. Given that altered patterning induced by Matrigel requires integrin α6β1 activity, it follows that laminin, rather than other factors associated with reconstituted ECM, is important for patterning early embryonic cells. This is further supported by the shared phenotype of *Itgb1^−/−^* and *Lamc1^−/−^* embryos.

In developing embryos or stem cell systems in which cells are yet to differentiate, myriad signals must be processed leading up to lineage commitment. During the first lineage segregation, Matrigel is sufficient to drive ICM specification in an integrin-dependent manner, irrespective of cell position. The ECM cues provided through Matrigel in our setup may be more concentrated than levels found *in vivo*, thereby overriding competing positional signals to drive ICM specification. Yet, integrin activity is not strictly required for initial inside-outside patterning *in vivo*. Given the significance of setting aside cells that will eventually form the embryo itself, other factors, such as the non-integrin laminin receptor dystroglycan, may be active in the embryonic interior as redundant ‘inside’ signals (Hynes, 1987; Mui et al., 2016; Williamson et al., 1997). Moreover, single cell gene expression data indicate that other integrins are also present during preimplantation development (Ohnishi et al., 2014). For example, integrins αvβ3 and αvβ5 are expressed alongside their cognate ECM ligand, vitronectin (Wayner et al., 1991). The precise contribution of individual ECM components and their receptors during development is a subject for future study.

Our work complements earlier studies in embryonic stem cells that revealed ECM-integrin signals as crucial regulators of the undifferentiated state and cell arrangement (Aumailley et al., 2000; Cattavarayane et al., 2015; Li et al., 2002). Given the ubiquity and tissue/stage-dependent complexity of the ECM and its receptors, their role in cell fate specification and pattern formation extends beyond early mouse development (Huang and Ingber, 2005; Humphrey et al., 2014; Walma and Yamada, 2020; Watt and Huck, 2013). Elucidation of the mechanistic contribution of ECM-receptor signals to patterning across diverse contexts will be instrumental to how we approach various disease states and design regenerative therapies in the future.

## MATERIALS AND METHODS

### Animal work

All animal work was performed in the Laboratory Animals Resources (LAR) facility at the European Molecular Biology Laboratory (EMBL) with permission from the Institutional Animal Care and Use Committee (IACUC) overseeing the operations (IACUC #TH110011). The LAR facility operates according to guidelines and recommendations set by the Federation for Laboratory Animal Science Associations. Mice were maintained in pathogen-free conditions under a 12-h light-12-h dark cycle.

### Mouse lines

WT mice were of a F1 hybrid strain from C57BL/6 and C3H (B6C3F1) animals. The following transgenic lines were used in this study: *Itgb1^tm1Efu (floxed)^* (Raghavan et al., 2000); *Lamc1^tmStrl (floxed)^* (Chen and Strickland, 2003); and *Zp3*-*Cre* (de Vries et al., 2000). Standard tail genotyping procedures were used to genotype the transgenic mice.

To obtain *Itgb1^+/−^* mice, *Itgb1^tm1Efu (floxed)^ Zp3-Cre^tg^* females were crossed with B6C3F1 males. To obtain zygotic *Itgb1^−/−^* embryos, *Itgb1*^+/−^ females were crossed with *Itgb1*^+/−^ males. To obtain *Lamc1^+/−^* mice, *Lamc1^tmStrl (floxed)^ Zp3-Cre^tg^* females were crossed with B6C3F1 males. To obtain zygotic *Lamc1^−/−^* embryos, *Lamc1*^+/−^ females were crossed with *Lamc1*^+/−^ males.

### Superovulation and dissection of reproductive organs

Superovulation was induced in females at 8-22 weeks of age prior to mating to increase the number of preimplantation embryos obtained per mouse. Intraperitoneal injection of 5 IU of PMSG (Intervet) and hCG (Intervet) were carried out, with a 48-50 h interval between the two injections. Immediately following hCG injection, each female mouse was put in a cage with a male for mating.

Timing of sacrifice post-hCG injection depended on the developmental stage relevant for the experiment. Hormone injections for superovulation were administered at 11:00 h and, thus, 16-32 cell stage embryos were recovered in the afternoon of E2.5. Early blastocysts were obtained on the morning of E3.5, and were then cultured overnight *in vitro* for the assessment of late blastocysts.

### Embryo work

Preimplantation embryos were obtained by flushing the oviduct from the infundibulum with a 1 ml syringe filled with HEPES (H)-KSOM (LifeGlobal, LGGH-050). All live embryos were handled under a Zeiss Discovery.v8 stereomicroscope equipped with a MATS-UST2 heating plate (Tokai Hit). All live embryos were cultured in 10 µl microdroplets of KSOM (Lawitts and Biggers, 1991) with a mineral oil (Sigma, M8410) overlay inside an Heracell 240i incubator (Thermo Fisher Scientific) with a 37°C humidified atmosphere of 5% CO_2_ and 95% air. Micromanipulations outside the incubator were carried out in H-KSOM.

### Immunosurgery

Zona pellucida were removed from embryos with 3-4 min pronase treatment [0.5% w/v proteinase K (Sigma-Aldrich, P8811) in H-KSOM supplemented with 0.5% PVP-40 (Sigma-Aldrich, P0930)] at 37°C. Subsequently, embryos were incubated in serum containing anti-mouse antibody (Cedarlane, CL2301; lot no. 049M4847V) diluted 1:3 with KSOM for 30 min at 37°C. Following three brief washes in H-KSOM, embryos were incubated in guinea pig complement (Sigma-Aldrich, 1639; lot no. SLBX9353) diluted 1:3 with KSOM for 30 min at 37°C. Lysed outer cells were removed by mouth-pipetting with a narrow glass capillary to isolate the inner cells.

### Embedding cells in Matrigel

The Matrigel mix consisted of Matrigel (Corning, 356230; lot no. 7345012) diluted in Dulbecco's PBS (DPBS; in house) to the desired concentration (4.5 mg/ml). Matrigel mix was prepared fresh for each experiment, mixed thoroughly through pipetting, and kept on ice during immunosurgery. Upon completion of immunosurgery, isolated inner cells were promptly resuspended in the Matrigel mix, and 15 µl droplets were added to 35 mm petri dishes (Falcon, 351008). To ensure that cell clusters from different embryos did not stick together, a closed glass capillary was used to space them apart. The petri dishes were inverted to prevent cells sticking to the bottom of the dish and then incubated at 37°C for 30 min for the mix to form a gel. After gel formation, 4 ml of prewarmed KSOM was gently pipetted into each dish to cover the gel.

To inhibit integrin heterodimer activity in Matrigel-embedded cells, blocking antibodies Ha2/5 and GoH3, which target integrin β1 and α6, respectively, were added to the overlying KSOM medium at a concentration of 10 µg/ml.

### Immunostaining

Embryos were fixed in 4% PFA (Sigma-Aldrich, P6148) at room temperature for 15 min, washed three times (5 min each) in wash buffer [DPBS-Tween (T) containing 2% bovine serum albumin (BSA; Sigma-Aldrich, A9647)], permeabilised at room temperature for 30 min in permeabilisation buffer (0.5% Triton-X in DPBS; Sigma-Aldrich, T8787), washed three times each for 5 min and then incubated in blocking buffer (PBS-T containing 5% BSA) either overnight at 4°C or for 2 h at room temperature. Blocked samples were incubated with primary antibodies (Table S2) overnight at 4°C, three times each for 5 min and then incubated in fluorophore-conjugated secondary antibodies and dyes (Table S3) for 2 h at room temperature. Stained samples were washed three times each for 5 min and then incubated in DAPI solution (Life Technologies, D3571; diluted 1:1000 in DPBS) for 10 min at room temperature. These samples were then transferred into droplets of DPBS overlaid with mineral oil on a 35 mm glass bottom dish (MatTek, P356-1.5-20-C) for imaging.

### Molecular work

#### Single embryo genotyping

Individual embryos were mouth pipetted into 200 µl PCR tubes containing 10 µl of lysis buffer consisting of 200 µg/ml Proteinase K in *Taq* polymerase buffer (Thermo Fischer Scientific, B38). The lysis reaction was left to occur for 1 h at 55°C, followed by 10 min at 96°C. The resulting genomic DNA was mixed with relevant primers (Table S4) for determination of genotype via PCR.

#### Microscopy and image analyses

Fixed and stained embryos were imaged on Zeiss LSM780 and LSM880 confocal microscopes. For both systems, a 40× water-immersion Zeiss C-Apochromat 1.2 NA objective lens was used. Imaging was carried out with the Zen (Zeiss) software interface. Resulting raw images were processed using ImageJ. Further quantification of fluorescence intensities and nuclei/cell counting were performed on either ImageJ or Imaris 9.2.1 (Oxford Instruments) as described below.

#### Measure of cell circularity

Circularity measurements were obtained by tracing the outline of individual cells on ImageJ following their membrane/actin signal. From each cluster, between four and eight TE- and ICM-specified cells were traced across their mid-section. Cells undergoing division were not included, because cells round up during division. Circularity on ImageJ is calculated using the following equation: *circularity=*4*π* (*area/perimeter*^2^).

#### Detection of nuclei and quantification of their spatial distribution and the fluorescence intensity of lineage markers

Imaris Surpass was used to detect nuclei and measure lineage specification because it allows 3D visualisation of the confocal image data. The ‘Add Spots’ function was used to detect each nucleus on the DAPI, CDX2, SOX2 or GATA4 channel. Estimated spot (nucleus) diameter was set to 6 µm, and manual corrections were made for each image as necessary to detect all nuclei. The mean fluorescence intensity of SOX2, CDX2 or GATA4 was measured for each detected nucleus. Spot detection of each nucleus was also used as a cell counter.

*x*-, *y*-, and *z*-coordinates were acquired for each nuclear spot from the statistics tab on Imaris, and used to measure the distance to the centre of the ICM. The ICM centre coordinates were acquired by calculating the mean between the maximum and minimum values among total ICM cells (EPI and PrE).

#### Quantification of fluorescence intensity of apicobasal markers

The fluorescence signal intensity of cortical pERM and integrin β1 was used as a measure of apical and basal polarity, respectively. Images of samples stained for these proteins were analysed on ImageJ. To reduce noise, the Gaussian filter was applied to smooth the image. For each *z*-stack, a mid-slice was selected, and a line was traced along the perimeter of the smoothed image or across cells/tissues of interest. A plot profile along the line was obtained for the pERM, PKCζ, or integrin β1 channel. Individual data points were exported from ImageJ for statistical analysis.

#### Segmentation of PrE and EPI tissue and subsequent sphericity measurement

Manual segmentation was performed in using the Surface function in Imaris Surpass. Based on fluorescence signals from cell fate markers (GATA4 for PrE and SOX2 for EPI) and membrane dye, PrE and EPI layers were traced for each *z*-stack of a confocal image acquired at 2 µm intervals. Automatic surface rendering on manual traces recreated PrE and EPI segments, and the sphericity of these 3D objects was calculated using the following equation:

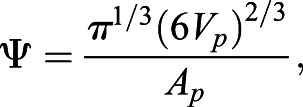
where Ψ is sphericity, V*p* is volume of the object and A*p* is surface area of the object.

#### Statistical analysis

Statistical analyses and graph generation were performed using the ggplot2 package in R and Microsoft Excel. Comparison of the distribution of fate marker intensities was performed by the Mann–Whitney *U*-test. Differences in cell count, surface enrichment of apicobasal polarity markers, circularity, ICM/cell sphericity and distance measurements were assessed using the Student's *t*-test (two-sided). Differences were significant at the P <0.05 level. The statistical relationships between EPI/PrE fate marker expression and cell position were assessed by Pearson's correlation. All statistical data are available in Table S1.

## Supplementary Material

Supplementary information

Reviewer comments
