# Supplementary material for: ECM-integrin signalling instructs cellular position sensing to pattern the early mouse embryo
Source: Development. 2022 Jan 13;149(1):dev200140. doi: 10.1242/dev.200140 (PMC8881741; doi:10.1242/dev.200140)
Supplement: Supplementary information [file develop-149-200140-s1.pdf]

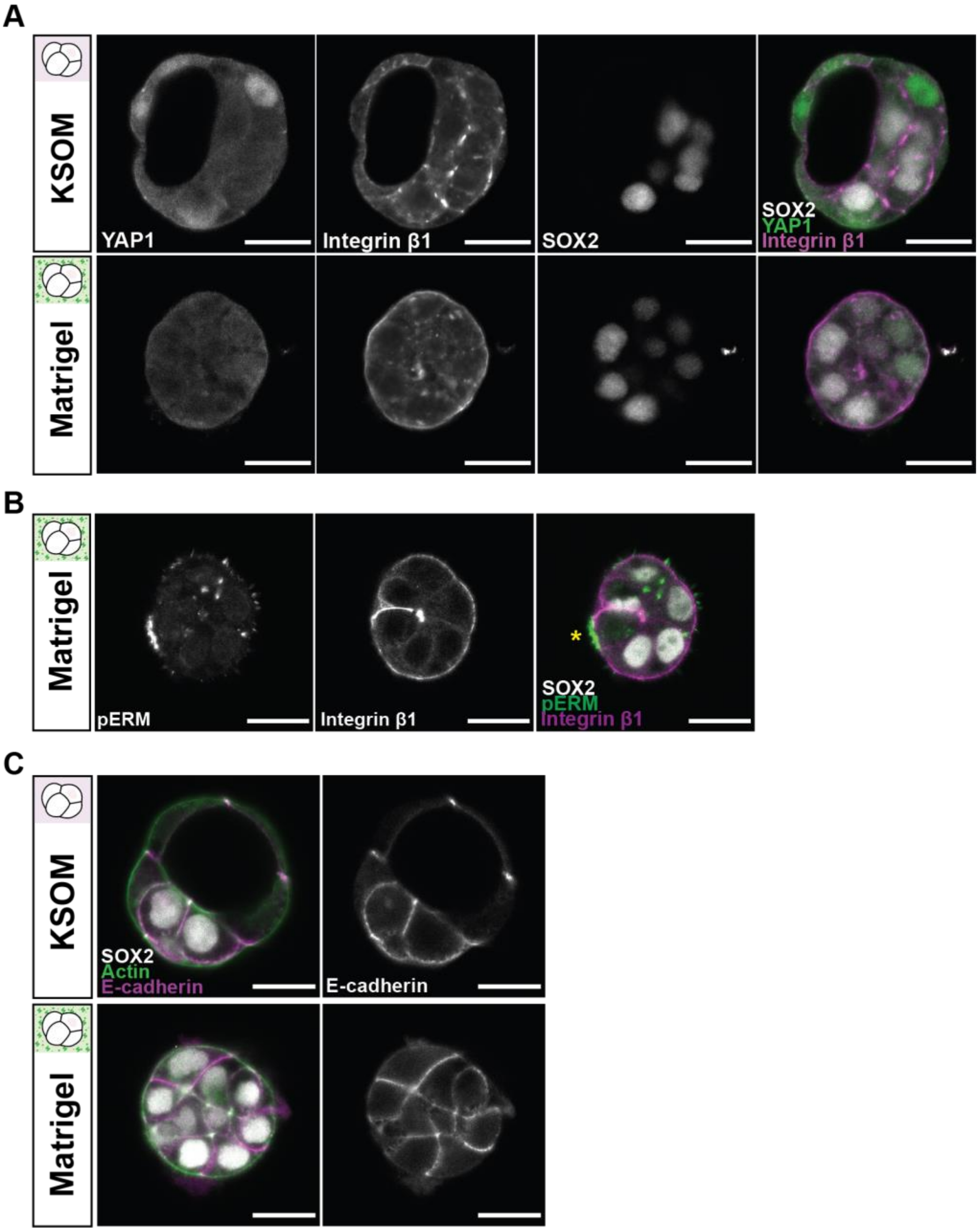

Fig. S1. Exogenous ECM drives Hippo signalling and suppresses apical polarity. Related to Figure 2. (A) Representative images of TE-ICM fate specification and integrin β1 distribution among cells following immunosurgery and culture in KSOM or Matrigel. SOX2 marks ICM fate while nuclear YAP1 is characteristic of TE cells.
(B) Partial enrichment of pERM on the surface of isolated cells cultured in Matrigel. The cell with the patch of pERM signal (*) is SOX2-negative.
(C) Representative images of E-cadherin localisation in isolated cells following culture in KSOM or Matrigel. Scale bars = 20 µm.

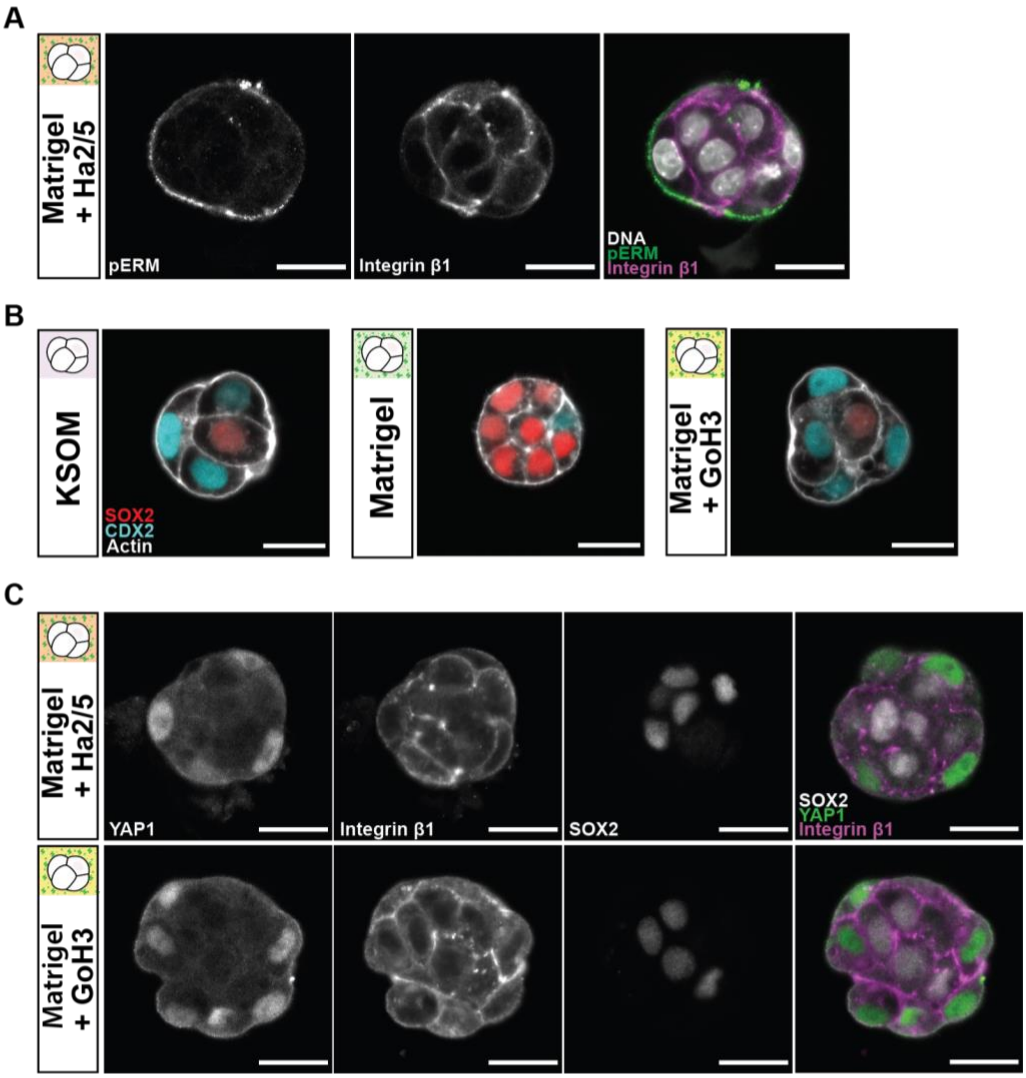

Fig. S2. Integrin α6β1 inhibition restores inside-outside patterning to Matrigel-cultured cells. Related to Figure 3. (A) Representative images of apicobasal polarity in cells cultured in Matrigel with integrin β1 function-blocking antibody Ha2/5 (10 μg/ml). Phosphorylated ERM (pERM) proteins mark apical domain.
(B) Representative images of TE-ICM fate specification following culture in KSOM, Matrigel, or Matrigel with integrin α6 function-blocking antibody GoH3 (10 μg/ml).
(C) Representative images of inside-outside patterning following culture in Matrigel with either Ha2/5 or GoH3. In addition to SOX2 expression, differential localisation of YAP1 distinguishes TE and ICM fate, as YAP1 is nuclear localised in TE cells. Scale bars = 20 μm.

Development • Supplementary information

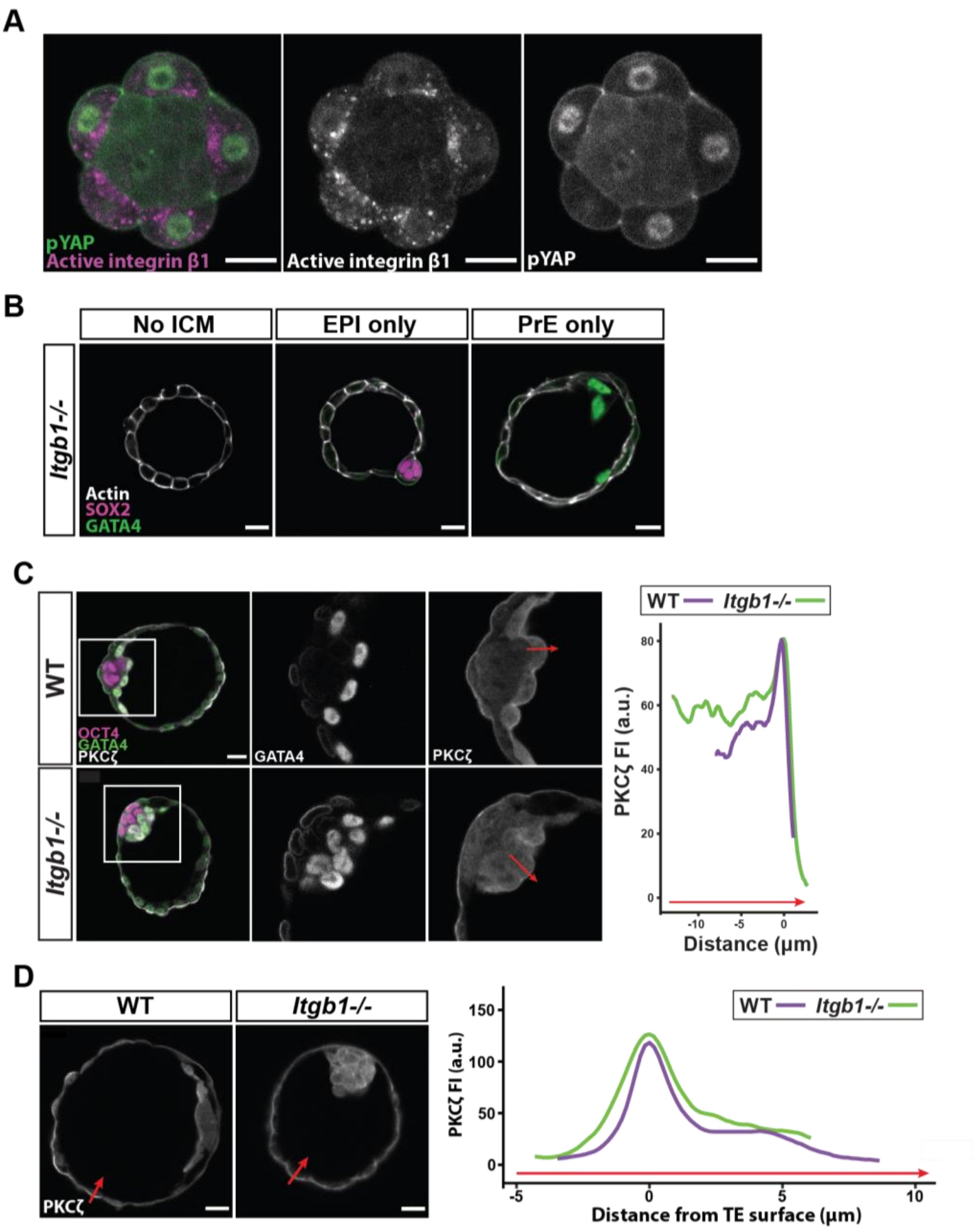

Fig. S3. EPI-PrE patterning in the late blastocyst *in vivo* requires integrin β1. Related to Figure 4.

(A) Representative images show localisation of the active conformation of integrin β1 (12G10 antibody) in the morula stage embryo. Phosphorylated YAP (pYAP) signal distinguishes inside and outside cells, as inside cells exhibit cytoplasmic pYAP localisation.

(B) Images of *Itgb1*$^{-/-}$ blastocysts with severe disruption of ICM.

(C) Representative images show PKCζ distribution across the PrE in WT and *Itgb1*$^{-/-}$ blastocysts at E4.0, followed by plot profile of fluorescence intensity along line of interest across the PrE layer (red arrow).

(D) Representative images of PKCζ distribution in WT and *Itgb1*$^{-/-}$ blastocysts at E4.0, followed by profile plot of fluorescence intensity along line of interest (red arrow) across the TE. Plot profiles are aligned based on the point of maximum PKCζ intensity at the apical surface of TE surface (distance "0").

Development • Supplementary information

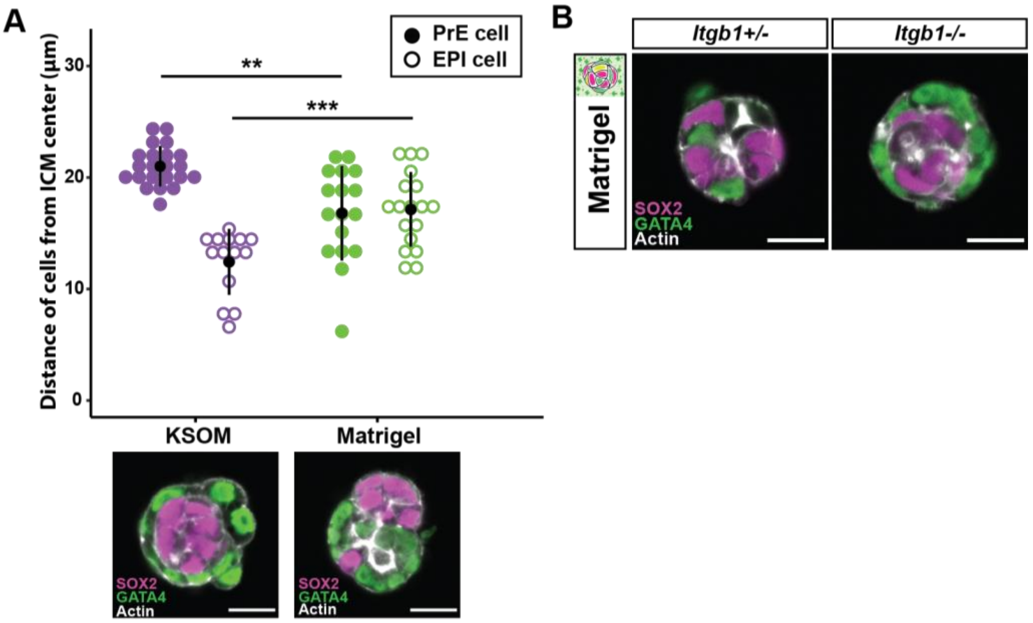

Fig. S4. Matrigel disrupts spatial arrangement of EPI/PrE cells in isolated ICMs. Related to Figure 5.

(A) Distance of PrE and EPI cells from the center of the ICM cultured in either KSOM or Matrigel. Distance data are from representative samples displayed beneath the plot (same as images from Figure 5D and 5F).

(B) Representative images of ICMs isolated from E3.5 *Itgb1* transgenic embryos and cultured in either Matrigel. ICMs from *Itgb1+/-* embryos serve as littermate controls. SOX2 marks EPI cells, and GATA4 marks PrE cells. Scale bars = 20 µm.

Development • Supplementary information

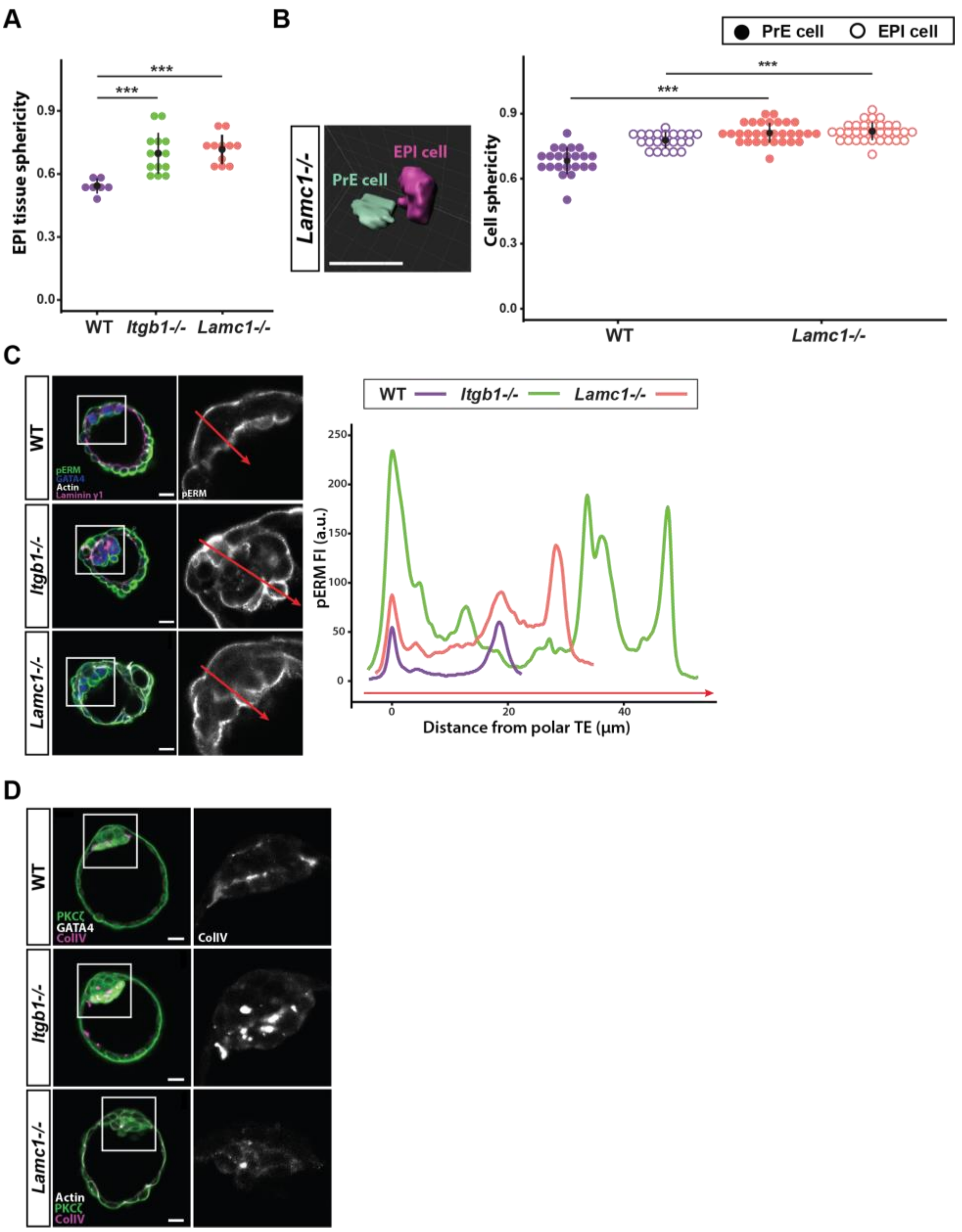

Fig. S5. *Lamc1-/-* embryos share phenotype with *Itgb1-/-* embryos at E4.0. Related to Figure 6.

(A) Sphericity of segmented EPI tissues from WT, *Itgb1-/-*, and *Lamc1-/-* blastocysts at E4.0. Error bars show mean ± s.d. Student's *t*-test, two-sided. N = 32 embryos. *** $p < 0.001$.

(B) Representative image of individual *Lamc1-/-* PrE and EPI cells segmented on Imaris. Dot plot displays sphericity measured from individual segmented surfaces. Error bars show mean ± s.d. Student's *t*-test, two-sided. N = 106 cells from 17 embryos. *** $p < 0.001$.

(C) Representative image of morphology and apical polarity of the ICM in WT, *Itgb1*$^{-/-}$, and *Lamc1-/-* blastocysts at E4.0. Accompanying intensity profile shows distribution of pERM across the ICM along the red line of interest. Data for WT and *Itgb1-/-* are duplicated from Figure 4G for ease of comparison with *Lamc1-/-* mutants.

(D) Representative images show distribution of basal collagen IV in WT, *Itgb1-/-* and *Lamc1-/-* embryos at E4.0. Scale bars = 20 μm.

Table S1. Primary data for statistical figures.

Click here to download Table S1

Development • Supplementary information

Table S2. Primary antibodies

Epitope	Host	Catalogue #	Company	Dilution
aPKC (PKCζ)	Rabbit	sc-216	Santa Cruz Biotechnology	1:200
CDX2	Mouse	MU392A-UC	Biogenex	1:200
E-cadherin	Rat	U3254	Sigma Aldrich	1:100
GATA4	Goat	AF2606	R&D Systems	1:200
GATA6	Goat	AF1700	R&D Systems	1:200
Integrin α6 (GoH3)	Rat	555734	BD Pharmingen	1:100
Integrin β1	Rat	MAB1997	Millipore	1:100
Integrin β1 (Ha2/5)	Rat	555002	BD Pharmingen	1:100
Integrin β1 (active, 9EG7)	Rat	553715	BD Pharmingen	1:100
Integrin β1 (active, 12G10)	Mouse	sc-59827	Santa Cruz Biotechnology	1:100
Laminin (non-chain specific)	Rabbit	NB300-14422	Novus Biologicals	1:100
Laminin α5	Rat	n/a	Gift from Lydia Sorokin	N/A
Laminin β1	Rat	n/a	Gift from Lydia Sorokin	N/A
Laminin γ1	Rat	n/a	Gift from Lydia Sorokin	N/A
NANOG	Rabbit	RCAB002P-F	ReproCELL, Inc	1:200
YAP1	Mouse	H00010413-M01	Abnova	1:100
Phospho-ERM	Rabbit	3726	Cell Signaling Technology	1:200
Sox-2 (D9B8N)	Rabbit	23064	Cell Signaling Technology	1:200

Table S3. Secondary antibodies and dyes

Fluorophore	Target	Host	Catalogue #	Company	Dilution
Alexa Fluor 488	Goat IgG	Donkey	A11055	Life Technologies	1:200
Alexa Fluor 488 Plus	Rabbit IgG	Donkey	A32790	ThermoFisher	1:200
Alexa Fluor 546	Rabbit IgG	Donkey	A10040	ThermoFisher	
Cy5	Mouse IgG	Donkey	715-175-150	Jackson ImmunoResearch	1:200
Cy5	Rat IgG	Donkey	712-175-153	Jackson ImmunoResearch	1:200
DAPI	(DNA)	-	D3571	Life Technologies	1:1000
Rhodamine phalloidin	(Actin)	-	R415	Invitrogen	1:200

Table S4. Sequence of genotyping primers

Mouse line/locus	Primer 1	Primer 2	Primer 3
Itgb1 deleted	TGAATATGGGCTTG GCAGTTA	CCACAACTTTCCCAG TTAGCTCTC	
Itgb1 tm1Efu (floxed)	CGGCTCAAAGCAG AGTGTCAGTC	CCACAACTTTCCCAG TTAGCTCTC	
Lamc1 deleted/ *Lamc1* tmStrl (floxed)	AAA GAA GCA GAG TGT GGG GG	TGG CCT TTT CAA CCC TGG AA	GCC TTC TAT CGC CTT CTT GAC
ZP3 Cre	TGCTGTTTCACTGG TTGTGCGGCG	TGCCTTCTCTACACC TGCGGTGCT	

Development • Supplementary information
