# Supplementary material for: ECM-integrin signalling instructs cellular position sensing to pattern the early mouse embryo
Source: Development. 2022 Jan 13;149(1):dev200140. doi: 10.1242/dev.200140 (PMC8881741; doi:10.1242/dev.200140)
Supplement: Reviewer comments [file develop-149-200140_review_history.pdf]

ECM-integrin signalling instructs cellular position-sensing to pattern the early mouse embryo

Esther Jeong Yoon Kim, Lydia Sorokin and Takashi Hiiragi
DOI: 10.1242/dev.200140

Editor: Patrick Tam

Review timeline
Original submission: 26 August 2021
Editorial decision: 20 September 2021
First revision received: 16 November 2021
Accepted: 3 December 2021

Original submission

First decision letter

MS ID#: DEVELOP/2021/200140

MS TITLE: ECM-integrin signalling instructs cellular position-sensing to pattern the early mouse embryo

AUTHORS: Esther Jeong Yoon Kim, Lydia Sorokin, and Takashi Hiiragi

I have now received all the referees' reports on the above manuscript, and have reached a decision. The referees' comments are appended below, or you can access them online: please go to BenchPress and click on the 'Manuscripts with Decisions' queue in the Author Area.

As you will see, the referees express considerable interest in your work, but have some significant criticisms of the design of the experiments for testing the action of integrin b1 in isolated ICMs, the segregation of epiblast and the 'abnormal'¤ primitive endoderm in the blastocyst and the inconsistency of the findings with the phenotype of Itgb1 and Lamc1 mutants. On these considerations, a substantial revision of the manuscript is recommended before we can consider publication. If you are able to revise the manuscript along the lines suggested, which may involve further experiments, I will be happy receive a revised version of the manuscript. Your revised paper will be re-reviewed by one or more of the original referees, and acceptance of your manuscript will depend on your addressing satisfactorily the reviewers' major concerns. Please also note that Development will normally permit only one round of major revision.

We are aware that you may be experiencing disruption to the normal running of your lab that make experimental revisions challenging. If it would be helpful, we encourage you to contact us to discuss your revision in greater detail. Please send us a point-by-point response indicating where you are able to address concerns raised (either experimentally or by changes to the text) and where you will not be able to do so within the normal timeframe of a revision. We will then provide further guidance. Please also note that we are happy to extend revision timeframes as necessary.

Please attend to all of the reviewers' comments and ensure that you clearly highlight all changes made in the revised manuscript. Please avoid using 'Tracked changes' in Word files as these are lost in PDF conversion. I should be grateful if you would also provide a point-by-point response detailing how you have dealt with the points raised by the reviewers in the 'Response to Reviewers' box. If you do not agree with any of their criticisms or suggestions please explain clearly why this is so.

© 2022. Published by The Company of Biologists under the terms of the Creative Commons Attribution License (https://creativecommons.org/licenses/by/4.0/).

Reviewer 1

Advance summary and potential significance to field

During preimplantation development, three types of cells are formed during the two rounds of cell differentiation. The first cell differentiation into TE and ICM is controlled by the Hippo signaling pathway, which is regulated by E-cadherin-mediated cell adhesion and cell polarity. The second differentiation of ICM into EPI and PrE cells is regulated by the FGF signaling pathway, the ligand of which is expressed in EPI cells.

This is a high-quality paper describing the potential roles of ECM-integrin signaling in the cell differentiation processes. The authors first demonstrated using live imaging that ICM specification occurs only after complete internalization of the cells. The authors also demonstrated that laminins and integrins are expressed at the cell-cell interface of the morula and ICM of blastocysts. In vitro culture of the inner cells isolated from the morula stage embryos in the laminin-rich ECM (Matrigel) promoted ICM specification of the outer cells in an integrin b1-dependent manner. However, the analyses of Itgb1-/- embryos revealed that integrin b1 is not required for ICM specification in vivo. In Itgb1-/- embryos, PrE cells failed to polarize and form a multilayer tissue. Culture of isolated ICM in the ECM promoted outer localization of EPI cells. Based on these results, the authors concluded that laminin-integrin signaling functions as an internal positional signal and is required for sorting EPI/PrE cells.

It is a novel finding that ECM-integrin signaling is involved in cell fate regulation as an internal positional cue. However, the evidence supporting the operation of the proposed underlying mechanism during ICM-TE specification is weak, and the conclusion that integrin signaling is required for sorting EPI/PrE cells is misleading.

Comments for the author

Major comments:
1. The major concern regarding this study is that it is not clear whether this mechanism actually operates during the ICM specification. Itgb1-/-and Lamc1-/- embryos did not exhibit defects in ICM specification, and the expression of laminin gamma1 at the morula stage was very weak. These observations raised the question of whether the laminin-integrin signaling is involved in ICM fate specification. Figure 7D shows that strong active integrin b1 signals were detected only at the interface of EPI and PrE cells, although integrin b1 was present in all cellular boundaries (Figure 6D), suggesting that the presence of integrin and laminin proteins does not necessarily indicate the activation of integrin signaling. Therefore, to demonstrate the operation of integrin signaling during ICM specification, the distribution of active integrin b1 should be examined together with the expression of ICM marker SOX2 in 16- and 32-cell stage embryos.

2. The authors concluded that integrin signaling is required for sorting EPI/PrE cells (stated in the Abstract). However, this conclusion is misleading because EPI and PrE cells were found to segregate properly in both Itgb1-/-and Lamc1-/- embryos. The abnormal phenotype of the mutants was that the PrE cells failed to form an epithelial monolayer. Therefore, integrin signaling is not required for cell sorting, but is required for the formation of PrE monolayer. Since the mutant PrE cells also failed to establish an apico-basal polarity, the failure in polarization or epithelialization is likely a primary defect.

Minor comments:
1. The information regarding the anti-active integrin b1 antibody is missing.
2. p15. Please remove the sentence “(Error! Reference source not found.)”.

Reviewer 2

Advance summary and potential significance to field

This manuscript describes studies with early mouse embryos ex vivo in which the role of ECM and integrins in specification and patterning are analyzed. The isolated inner regenerates the TE layer

© 2022. Published by The Company of Biologists under the terms of the Creative Commons Attribution License (https://creativecommons.org/licenses/by/4.0/).

in medium, whereas addition of matrigel blocks this process and favors ICM cell fate, which requires integrin a6b1.
Integrins also modulate specification of PrE vs EPI cell fate in the ICM.

Overall, these are well done experiments with results presented clearly. The manuscript presents new information that will be of interest to developmental biologists. The one major drawback is that deleting or blocking integrin b1 has very modest effects under normal conditions as opposed to the artificial setting of stripped ICM implanted in matrigel. Some further exploration of this discrepancy is needed to enhance the significance of the work.

Comments for the author

Fig 4. The finding that blocking or deletion of integrin a6b1 prevents the effects of matrigel but the effects on normal development are slight raises the question do other integrins replace b1 or are the effects of matrigel in the manipulated embryo artificial. This issue could be resolved by addressing whether other integrins are expressed and act in parallel to a6b1. Integrins a6b4 or avb3 are obvious candidates.
Fig 7. Talin is required for integrin activation and ligand binding, thus, it may be considered upstream as well as downstream. In any case, co-localization is not sufficient to conclude that "Integrin signalling is mediated by laminin and talin". The authors are probably better off backing away from this conclusion.

Minor:
Fig 1C, D X axis labels are missing or cut off.

Reviewer 3

Advance summary and potential significance to field

In their manuscript Kim and colleagues investigated the role of integrins (specifically of integrin β1 activity) in lineage specification and pattern formation in pre-implantation mouse embryos. In a convincing way, the authors demonstrated that that embryonic cells recognize their position within the embryo through the extracellular matrix (ECM) and integrin-mediated adhesion. Isolated early inner cells cultured in Matrigel, which is rich in laminin 111, did not form normal blastocysts but most of the cells in aggregates acquired ICM characteristics. Culture in Matrigel with the addition of Ha2/5 antibody that blocks integrin β1 function, resulted in restoration of wt phenotype. A similar effect was achieved via inhibition of integrin α6 activity. Furthermore, upon genetic ablation of Itgb1, integrin β1-deficient cells became refractory to the effects of Matrigel. At the same time PrE cells failed to resolve into a single monolayer in Itgb1-/- embryos.

Comments for the author

Kim and colleagues presented a series of intriguing observations that can definitely be of interest for scientists working on pre-implantation development as well as for the wider scientific community. However, not all of the conclusions reached by the authors are justified by the presented data.

Major points
1. Mutant Itgb1-/- and Lamc1-/- embryos undergo both TE and ICM as well as PrE and Epi specification and form blastocyst with all three lineages in appropriate position, which goes against the main title of the manuscript.
Both mutants present the same phenotype, where the shape of ICM and formation of PrE epithelium seems to be severely affected. However, in the manuscript the authors claim that both mutations alter the spatial arrangement between Epi and PrE cells. At the same time, on the images provided, the position of both Epi and PrE seem to be similar to the control embryos (Fig 5A and 7B). The main difference visible in the figures is in the 3D pattern of PrE cells that can be related to a possible disruption of PrE epithelium and/or to the problem with migration of parietal endoderm cells that in naive embryos originate from PrE. With this in mind, I would encourage the authors to look in more detail at the formation of TE and PrE epithelium in both mutants (Itgb1-/-

© 2022. Published by The Company of Biologists under the terms of the Creative Commons Attribution License (https://creativecommons.org/licenses/by/4.0/).

and Lamc1-/-) as this seems to be the main observable phenotype. It would be interesting to test whether indeed both epithelia are affected or the effect is restricted to PrE. Perhaps investigating the pattern of aPKC localization (and/or other polarity markers related to both TE and PrE) could better explain the observed phenotype. On a similar note, more in depth (preferably numerical) analysis of the 3D distribution of PrE cells in mutant embryo would strengthen the manuscript (from the presented images, PrE cells look much more packed and less organized in mutant embryos, despite being fully sorted). Another potential line of enquiry is to investigate whether the presented phenotype is not related to problems with cell migration (as presumptive parietal endoderm cells in wt embryos clearly imitated migration, that is absent in mutant embryos)

2. The data presented in figure 1 do not contribute anything to the rest of the manuscript. Although elegantly presented, the data presented in figure 1 do not have any clear link to the rest of the manuscript.
This impression is further deepened by the complete omission of the data presented in this figure from the discussion. It seems therefore that even the authors were not sure how to connect the data from figure 1 with the main topic of the manuscript. I would strongly recommend removing this part, especially that using GFP expression as a proxy of the final abundance of SOX2 protein in the cells is not fully justified.
3. The interpretation of the data presented in figure 5C and D is not convincing.
Despite some differences in the level of aPKC signal, the general trend presented on figure 5D seems to be the same for wt and mutant embryos. More detailed analysis of polarisation in mutant and wt embryos would strengthen the manuscript.
4. The data from figure 6F seems to point towards a very interesting phenomenon.
Matrigel-treated salt and pepper ICMs seem to be able to separate PrE and EPI cells into two different compartments. However, the special arrangement of these cells is very different than embryos cultured in Matrigel with Ha2/5. Are the images presented in this figure representative for the whole cohort of embryos? If yes, this would suggest that although cell sorting still occurs in Matrigel-only culture, the 3D arrangement of cells is severely disrupted. This could potentially (if properly measured) strengthen the authors claim that 3D structure of PrE and Epi layers may be disrupted upon exposing cells to laminin 111. Why this does not happen in the mutant embryos will also need further explanation.

Minor points:
More info about how cell circularity was measured would be beneficial

---

First revision

Author response to reviewers' comments

Response to Reviewers

We thank all reviewers for their insightful and constructive feedback. By performing additional experiments and analyses, we have addressed the major and minor comments below.

Reviewer #1
During preimplantation development, three types of cells are formed during the two rounds of cell differentiation. The first cell differentiation into TE and ICM is controlled by the Hippo signaling pathway, which is regulated by E-cadherin-mediated cell adhesion and cell polarity. The second differentiation of ICM into EPI and PrE cells is regulated by the FGF signaling pathway, the ligand of which is expressed in EPI cells.

This is a high-quality paper describing the potential roles of ECM-integrin signaling in the cell differentiation processes. The authors first demonstrated using live imaging that ICM specification occurs only after complete internalization of the cells. The authors also demonstrated that laminins and integrins are expressed at the cell-cell interface of the morula and ICM of blastocysts. In vitro culture of the inner cells isolated from the morula stage embryos in the laminin-rich ECM (Matrigel) promoted ICM specification of the outer cells in an integrin b1-

© 2022. Published by The Company of Biologists under the terms of the Creative Commons Attribution License (https://creativecommons.org/licenses/by/4.0/).

dependent manner. However, the analyses of Itgb1-/- embryos revealed that integrin b1 is not required for ICM specification in vivo. In Itgb1-/- embryos, PrE cells failed to polarize and form a multilayer tissue. Culture of isolated ICM in the ECM promoted outer localization of EPI cells. Based on these results, the authors concluded that laminin-integrin signaling functions as an internal positional signal and is required for sorting EPI/PrE cells.

It is a novel finding that ECM-integrin signaling is involved in cell fate regulation as an internal positional cue. However, the evidence supporting the operation of the proposed underlying mechanism during ICM-TE specification is weak, and the conclusion that integrin signaling is required for sorting EPI/PrE cells is misleading.

Major comments:

1. The major concern regarding this study is that it is not clear whether this mechanism actually operates during the ICM specification. Itgb1-/-and Lamc1-/- embryos did not exhibit defects in ICM specification, and the expression of laminin gamma1 at the morula stage was very weak. These observations raised the question of whether the laminin-integrin signaling is involved in ICM fate specification. Figure 7D shows that strong active integrin b1 signals were detected only at the interface of EPI and PrE cells, although integrin b1 was present in all cellular boundaries (Figure 6D), suggesting that the presence of integrin and laminin proteins does not necessarily indicate the activation of integrin signaling.
Therefore, to demonstrate the operation of integrin signaling during ICM specification, the distribution of active integrin b1 should be examined together with the expression of ICM marker SOX2 in 16- and 32-cell stage embryos.

The reviewer is correct to point out that the presence of integrin and laminin does not necessarily indicate active signalling. Therefore, we used a conformation-specific antibody for integrin β1 to examine signalling (Bazzoni et al., 1995; Humphries et al., 2005). The active conformation of integrin β1 (12G10 antibody) is found on the basal side of outer cells in the 16-32 cell morula, away from the apical surface (Response Figure 1). This suggests that initial ICM specification does not involve active integrin signalling within inner cells *in vivo*. As pointed out by the reviewer, this is consistent with the phenotype of *Itgb1-/-* and *Lamc1-/-* mutants. We added these data as Figure S3A and discussed it accordingly in the text (highlighted).

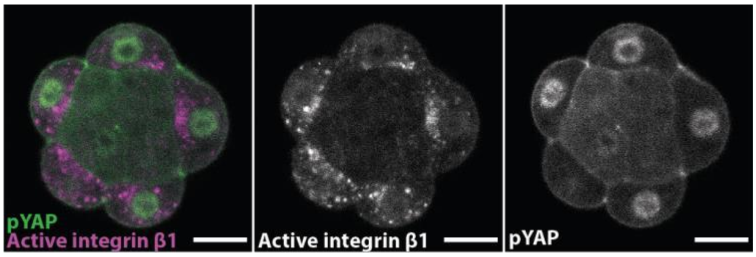

Response Figure 1. Integrin β1 is active in outer cells of the morula.
Representative images show localisation of the active conformation of integrin β1 (12G10 antibody) in the morula stage embryo. Inside cells are distinguishable by cytoplasmic localisation of phosphorylated YAP (pYAP) protein (Maître et al., 2016), as *Sox2* expression is generally low during this stage. Scale bars = 20 µm.

2. The authors concluded that integrin signaling is required for sorting EPI/PrE cells (stated in the Abstract). However, this conclusion is misleading because EPI and PrE cells were found to segregate properly in both Itgb1-/-and Lamc1-/- embryos. The abnormal phenotype of the mutants was that the PrE cells failed to form an epithelial monolayer. Therefore, integrin signaling is not required for cell sorting, but is required for the formation of PrE monolayer. Since the mutant PrE cells also failed to establish an apico-basal polarity, the failure in polarization or epithelialization is likely a primary defect.

We fully agree with the reviewer that the major defect in *Itgb1-/-* and *Lamc1-/-* embryos is the failure to form an epithelial PrE monolayer, rather than sorting between EPI and PrE lineages. Segmentation of the two ICM lineages revealed increased sphericity of the PrE tissue in mutants

© 2022. Published by The Company of Biologists under the terms of the Creative Commons Attribution License (https://creativecommons.org/licenses/by/4.0/).

due to failure of PrE cells to spread out in a monolayer (Response Figure 2A). This was further supported by the spatial distribution of PrE cells, which were clustered more closely to the ICM center in *Itgb1-/-* and *Lamc1-/-* blastocysts compared to wildtype (WT) (Response Figure 2B).

Furthermore, we examined the distribution pattern of phosphorylated ERM (ezrin, radixin, moesin, henceforth pERM) proteins to assess apical polarity in greater detail, in addition to the existing PKCζ data in the original text. While WT embryos exhibit bimodal pERM distribution with fluorescence intensity peaking at the surface of the polar TE and PrE, *Itgb1-/-* and *Lamc1-/-* profiles exhibit multiple peaks (Response Figure 2C). In addition, basal distribution of collagen IV appears more punctate in both mutants, while the protein is linearly organised on the basal side of the PrE in WT blastocysts (Response Figure 2D).
Similar reduction in PrE basal integrin β1 activity is also observed in *Lamc1-/-* mutants (Response Figure 2E). Therefore, both apical and basal markers examined show patchier and broader domains of localisation in *Itgb1-/-* and *Lamc1-/-* mutants compared to WT counterparts. These demonstrate that mutant embryos fail to form a PrE monolayer, and that their PrE tissues exhibit disrupted apical and basal surfaces. These descriptions have been added as Figures 4C-G, 6D-F, S5, and are discussed in the main text accordingly (highlighted).

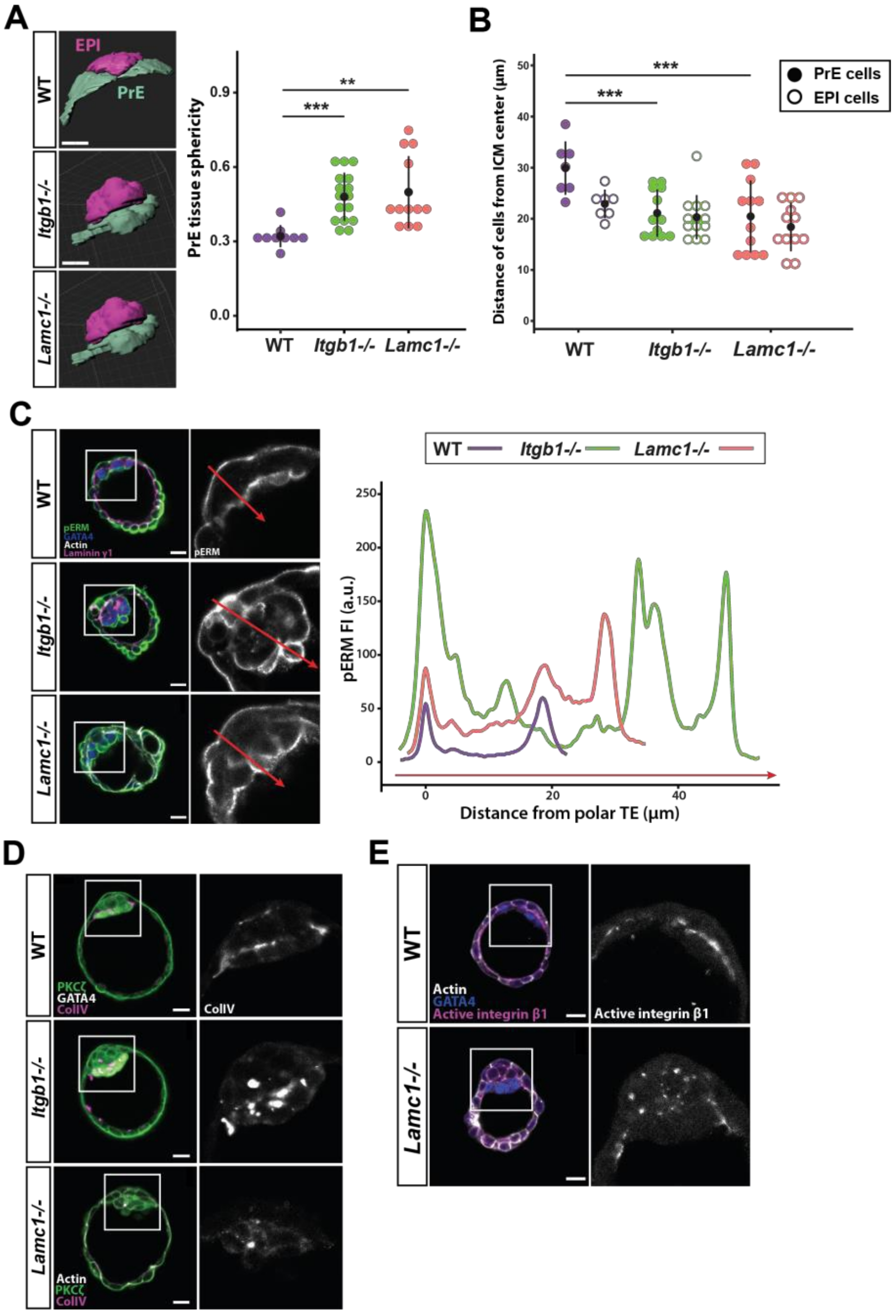

© 2022. Published by The Company of Biologists under the terms of the Creative Commons Attribution License (https://creativecommons.org/licenses/by/4.0/).

Response Figure 2. Apicobasal polarity of the PrE is disrupted in *Itgb1-/-* and *Lamc1-/-* embryos.
(A) Representative images of segmented PrE and EPI on Imaris. Sphericity of PrE tissue are compared across genotypes. Student's *t*-test, two-sided. Error bars show mean per embryo ± s.d. N = 37 embryos. ** $p < 0.01$, *** $p < 0.001$. (B) Mean distance of each nucleus from the center of the ICM is compared across genotypes. Each dot represents average value from all PrE or EPI cells in one embryo. Student's *t*-test, two-sided. Error bars show mean per embryo ± s.e.m. N = 32 embryos.
*** $p < 0.001$. (C) Left side: representative images show distribution of pERM across the inner cell mass (ICM) of WT, *Itgb1-/-*, and *Lamc1-/-* embryos at E4.0. Right side: fluorescence intensity profile of line of interest across the polar trophectoderm (TE) and ICM demonstrate quantitative differences in pERM distribution. Plot profiles are aligned based on the point of maximum pERM intensity at the polar TE surface (distance "0"). (D) Representative images show distribution of basal collagen IV in WT, *Itgb1-/-* and *Lamc1-/-* embryos at E4.0. (E) Representative images show distribution of active integrin β1(9EG7 antibody) in WT and *Lamc1-/-* mutant embryos at E4.0. Scale bars = 20 μm.
Minor comments:

1. *The information regarding the anti-active integrin b1 antibody is missing.*

This information has been added to the Methods section (Table 1) (highlighted).

2. *p15. Please remove the sentence "(Error! Reference source not found.)".*

This sentence has been removed.

Reviewer #2
This manuscript describes studies with early mouse embryos ex vivo in which the role of ECM and integrins in specification and patterning are analyzed. The isolated inner regenerates the TE layer in medium, whereas addition of matrigel blocks this process and favors ICM cell fate, which requires integrin a6b1. Integrins also modulate specification of PrE vs EPI cell fate in the ICM.

Overall, these are well done experiments with results presented clearly. The manuscript presents new information that will be of interest to developmental biologists. The one major drawback is that deleting or blocking integrin b1 has very modest effects under normal conditions as opposed to the artificial setting of stripped ICM implanted in matrigel. Some further exploration of this discrepancy is needed to enhance the significance of the work.

Major comments:
1. *Fig 4. The finding that blocking or deletion of integrin a6b1 prevents the effects of matrigel but the effects on normal development are slight raises the question, do other integrins replace b1 or are the effects of matrigel in the manipulated embryo artificial. This issue could be resolved by addressing whether other integrins are expressed and act in parallel to a6b1. Integrins a6b4 or avb3 are obvious candidates.*

The reviewer raises an excellent point, as it is certainly possible that other integrins contribute to preimplantation development. Examination of our single cell gene expression data (Ohnishi et al., 2014) indicates expression of integrins a3 (*Itga3*), av (*Itgav*), b3 (*Itgb3*) and b5 (*Itgb5*) during this stage (Response Figure 3A). Similar to integrin α6, the α3 subunit can heterodimerise with integrin β1 to recognise laminin (Gonzales et al., 1999). Moreover, just as the reviewer predicted, integrins av, b3, and b5 are also upregulated, alongside the cognate ECM ligand of αvβ3 and αvβ5 heterodimers, vitronectin (*Vtn*) (Wayner et al., 1991) (Response Figure 3B). The potential contribution of these additional integrins are discussed in the discussion section of the text (highlighted).

NOTE: We have removed unpublished data that had been provided for the referees in confidence.

© 2022. Published by The Company of Biologists under the terms of the Creative Commons Attribution License (https://creativecommons.org/licenses/by/4.0/).

2. Fig 7. Talin is required for integrin activation and ligand binding, thus, it may be considered upstream as well as downstream. In any case, co-localization is not sufficient to conclude that "Integrin signalling is mediated by laminin and talin". The authors are probably better off backing away from this conclusion.

We thank the reviewer for this insightful comment. The text has been modified accordingly to clear away from strong conclusions about the role of talin (highlighted).

Minor comments:

Fig 1C, D X axis labels are missing or cut off.

Figure 1 has been removed from the manuscript, in accordance to the suggestion by Reviewer #3.

Reviewer #3
In their manuscript Kim and colleagues investigated the role of integrins (specifically of integrin β1 activity) in lineage specification and pattern formation in pre-implantation mouse embryos. In a convincing way, the authors demonstrated that that embryonic cells recognize their position within the embryo through the extracellular matrix (ECM) and integrin-mediated adhesion. Isolated early inner cells cultured in Matrigel, which is rich in laminin 111, did not form normal blastocysts but most of the cells in aggregates acquired ICM characteristics.
Culture in Matrigel with the addition of Ha2/5 antibody that blocks integrin β1 function, resulted in restoration of wt phenotype. A similar effect was achieved via inhibition of integrin α6 activity. Furthermore, upon genetic ablation of Itgb1, integrin β1-deficient cells became refractory to the effects of Matrigel. At the same time PrE cells failed to resolve into a single monolayer in Itgb1-/- embryos.

Kim and colleagues presented a series of intriguing observations that can definitely be of interest for scientists working on pre-implantation development as well as for the wider scientific community. However, not all of the conclusions reached by the authors are justified by the presented data.

Major comments:

1. Mutant Itgb1-/- and Lamc1-/- embryos undergo both TE and ICM as well as PrE and Epi specification and form blastocyst with all three lineages in appropriate position, which goes against the main title of the manuscript.
Both mutants present the same phenotype, where the shape of ICM and formation of PrE epithelium seems to be severely affected. However, in the manuscript the authors claim that both mutations alter the spatial arrangement between Epi and PrE cells. At the same time, on the images provided, the position of both Epi and PrE seem to be similar to the control embryos (Fig 5A and 7B). The main difference visible in the figures is in the 3D pattern of PrE cells that can be related to a possible disruption of PrE epithelium and/or to the problem with migration of parietal endoderm cells that in naive embryos originate from PrE. With this in mind, I would encourage the authors to look in more detail at the formation of TE and PrE epithelium in both mutants (Itgb1-/- and Lamc1-/-) as this seems to be the main observable phenotype. It would be interesting to test whether indeed both epithelia are affected or the effect is restricted to PrE. Perhaps investigating the pattern of aPKC localization (and/or other polarity markers related to both TE and PrE) could better explain the observed phenotype. On a similar note, more in depth (preferably numerical) analysis of the 3D distribution of PrE cells in mutant embryo would strengthen the manuscript (from the presented images, PrE cells look much more packed and less organized in mutant embryos, despite being fully sorted). Another potential line of enquiry is to investigate whether the presented phenotype is not related to problems with cell migration (as presumptive parietal endoderm cells in wt embryos clearly imitated migration, that is absent in mutant embryos)

The reviewer's concerns are well-founded, and we agree that these warrant closer examination. Although EPI and PrE cells sort and their respective numbers average out to be comparable between WT and *Itgb1-/-* mutants, this is not always the case when comparing

© 2022. Published by The Company of Biologists under the terms of the Creative Commons Attribution License (https://creativecommons.org/licenses/by/4.0/).

individual embryos. While the most consistent *Itgb1-/-* phenotype is the failure to form an epithelial PrE monolayer, a few embryos exhibit more severe disruption of the ICM (Response Figure 4A). These include drastic reduction of ICM cell number, and skewing of the ratio of EPI/PrE cells. Furthermore, although the three lineages are in their appropriate positions at the tissue level as the reviewer pointed out, mispositioning of individual PrE cells are observed in mutants, as discussed below.

Localisation of apical marker, PKCζ, in the TE were comparable across WT and mutant embryos (Response Figure 4B), unlike in the PrE. This is further reinforced by the observation that mutant embryos develop fluid-filled cavities, which cannot form in the absence of a properly polarised TE epithelium.

For in-depth analysis of PrE distribution and morphology in 3D, we acquired *xyz* coordinates of each individual PrE and EPI nucleus using Imaris (Response Figure 4C), and measured its distance from the center of the ICM. In *Itgb1-/-* and *Lamc1-/-* mutants, PrE cells are less spread and clustered more closely to the ICM center as compared to WT embryos (Response Figure 4D). This is consistent with their more spherical morphology, which is apparent at the tissue level as well as in individual PrE cells, based on our segmentation on Imaris (Response Figure 4E-F). Together these demonstrate that, despite sorting of EPI and PrE cells, the overall morphology of the ICM and spatial arrangement of cells are disrupted in the absence of integrin. Due to failure to form an epithelial monolayer, some mutant PrE cells are found in the ICM interior rather than positioned adjacent to the fluid-filled cavity. In contast, the TE appears to be little affected. We have added these observations as Figures 4C-F, 6C-F, S3B and S3D, and added descriptions in the text (highlighted).

The question of cell migration raised by the reviewer is an astute one and an excellent base for future experiments. Others have indeed highlighted the importance of PrE migration in ICM patterning in WT (Wigger et al., 2017), but it is unfortunately not feasible to pursue this line of investigation given our current resources.

© 2022. Published by The Company of Biologists under the terms of the Creative Commons Attribution License (https://creativecommons.org/licenses/by/4.0/).

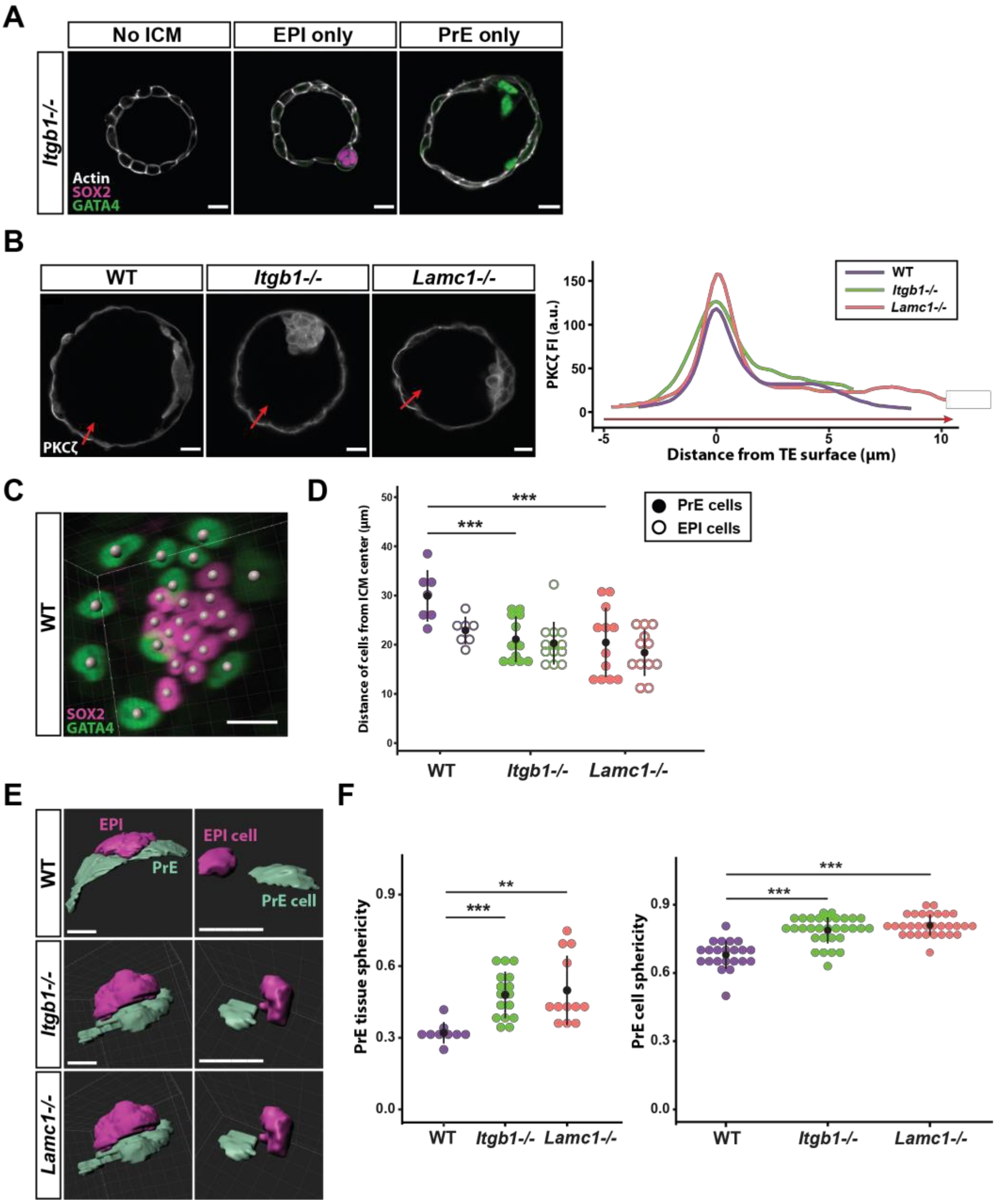

Response Figure 4. Integrin signalling is required for epithelial morphology of the PrE.
(A) Images of *Itgb1-/-* blastocysts with severe disruption of the ICM. (B) Representative images of PKCζ distribution in WT and mutant embryos at E4.0, followed by profile plot of fluorescence intensity along line of interest (red arrow) across the TE. Plot profiles are aligned based on the point of maximum PKCζ intensity at the apical TE surface (distance "0"). (C) Representative image of EPI(SOX2) and PrE(GATA4) nuclei detected in 3D on Imaris to acquire their spatial coordinates on Imaris. (D) Mean distance of each nucleus from the center of the ICM is compared across genotypes. Each dot represents average value from all PrE or EPI cells from one embryo. N = 32 embryos. (E) Representative images of segmented PrE and EPI, as well as individual segmented cells, on Imaris.
(F) Sphericity of PrE tissue (left), and individual PrE cells (right), are compared across genotypes. N = 37 embryos and 83 PrE cells. Student's *t*-test, ** $p < 0.01$, *** $p < 0.001$ Scale bars = 20 μm.

2. The data presented in figure 1 do not contribute anything to the rest of the manuscript. Although elegantly presented, the data presented in figure 1 do not have any clear link to the rest of the manuscript.
This impression is further deepened by the complete omission of the data presented in this figure from the discussion. It seems therefore that even the authors were not sure how to connect the

© 2022. Published by The Company of Biologists under the terms of the Creative Commons Attribution License (https://creativecommons.org/licenses/by/4.0/).

data from figure 1 with the main topic of the manuscript. I would strongly recommend removing this part, especially that using GFP expression as a proxy of the final abundance of SOX2 protein in the cells is not fully justified.

The reviewer’s criticisms are well-founded, and we have accordingly removed Figure 1 and the accompanying supplementary figure S1 from the manuscript. The importance of inside- positioning of cells for ICM specification is now described at the end of the Introduction (highlighted).

3. The interpretation of the data presented in figure 5C and D is not convincing. Despite some differences in the level of aPKC signal, the general trend presented on figure 5D seems to be the same for wt and mutant embryos. More detailed analysis of polarisation in mutant and wt embryos would strengthen the manuscript.

In addition to the aforementioned PKCζ distribution (Response Figure 5A), we used another apical marker, phosphorylated ERM (pERM) proteins, to assess polarity in *Itgb1-/-* mutants (Response Figure 5B). While WT embryos exhibit bimodal pERM distribution with fluorescence intensity peaking at the surface of the polar TE and PrE, *Itgb1-/-* profiles exhibit multiple peaks, indicating disrupted apical polarisation. These data show that polarity proteins are not restricted to the PrE apical membrane in the mutants. The distribution of pERM has been added as Figure 4G and is discussed accordingly in the text (highlighted).

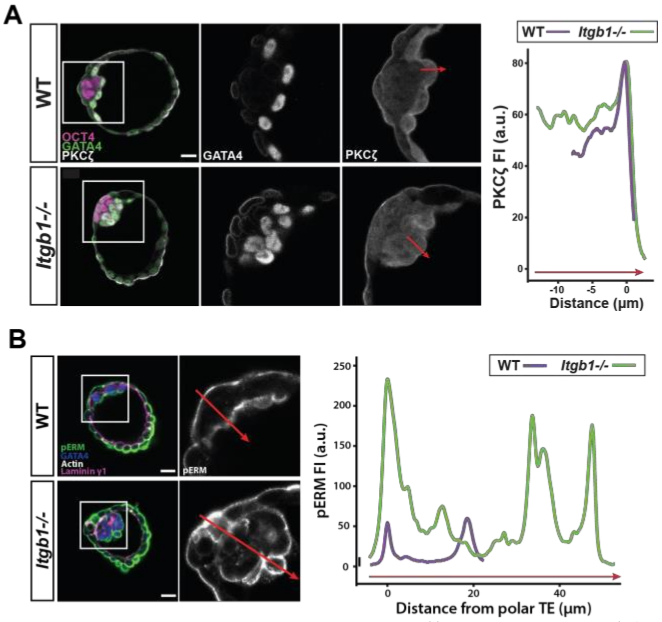

Response Figure 5. Integrin signalling is required for PrE polarity.
(A) Representative images show PKCζ distribution across the PrE in WT and *Itgb1-/-* embryos at E4.0, followed by plot profile of fluorescence intensity along line of interest across the PrE layer (red arrow). (B) Left side: representative images show distribution of pERM across the inner cell mass (ICM) of WT and *Itgb1-/-* embryos at E4.0. Right side: fluorescence intensity profile of line of interest across the polar trophectoderm (TE) and ICM demonstrate quantitative differences in pERM distribution. Plot profiles are aligned based on the point of maximum pERM intensity at the polar TE surface (distance “0”). Scale bars = 20 μm.

4. The data from figure 6F seems to point towards a very interesting phenomenon. Matrigel-treated salt and pepper ICMs seem to be able to separate PrE and EPI cells into two different compartments. However, the special arrangement of these cells is very different than embryos cultured in Matrigel with Ha2/5. Are the images presented in this figure representative for the whole cohort of embryos? If yes, this would suggest that although cell sorting still occurs in Matrigel-only culture, the 3D arrangement of cells is severely disrupted. This could potentially (if properly measured) strengthen the authors claim that 3D structure of PrE and Epi layers may be disrupted upon exposing cells to laminin 111. Why this does not happen in the mutant embryos will also need further explanation.

© 2022. Published by The Company of Biologists under the terms of the Creative Commons Attribution License (https://creativecommons.org/licenses/by/4.0/).

The reviewer raises an important point regarding the 3D structure of PrE and EPI cells exposed to Matrigel. The images presented in the pre-revision Figure 6F are representative of the cohort of samples, and additional images are presented below (Response Figure 6A). As the reviewer pointed out, PrE and EPI cells generally sort in Matrigel, though this is not always the case (upper right panel, Response Figure 6A). The disrupted distribution of cells in Matrigel as compared to KSOM control is quantified by the distance of cells from the center of the cultured ICM (Response Figure 6B). In KSOM, PrE cells are further from the ICM center compared to EPI cells due to their outside and inside positioning, respectively.

In Matrigel, PrE cells are closer and EPI cells are further from the center on average compared to their counterparts in KSOM, such that spatial position cannot distinguish the two lineages.

In contrast, ICMs isolated from *Itgb1-/-* embryos are refractory to the effects of Matrigel, and PrE cells surround EPI cells (Response Figure 6C). This resembles the effects of integrin β1-blocking antibody Ha2/5 (bottom panels, Response Figure 6A). These show that in Matrigel, integrin β1 is required to relay signals from laminin to affect PrE/EPI patterning.

While Matrigel culture provides laminin all around the isolated ICM, endogenous laminin-integrin activity is disrupted at the EPI-PrE boundary in *Itgb1-/-* blastocysts. In both cases, EPI/PrE sorting occurs, but we observe shared disruption of polarity and 3D patterning as described earlier (Response Figures 4D-F, 5, 6A and 6B). These consistently demonstrate that integrin signalling provides positional cues that underlie embryonic patterning of the ICM. Response figures 6B and 6C have been added as Figures S4A and S4B, respectively, and are discussed accordingly in the text (highlighted).

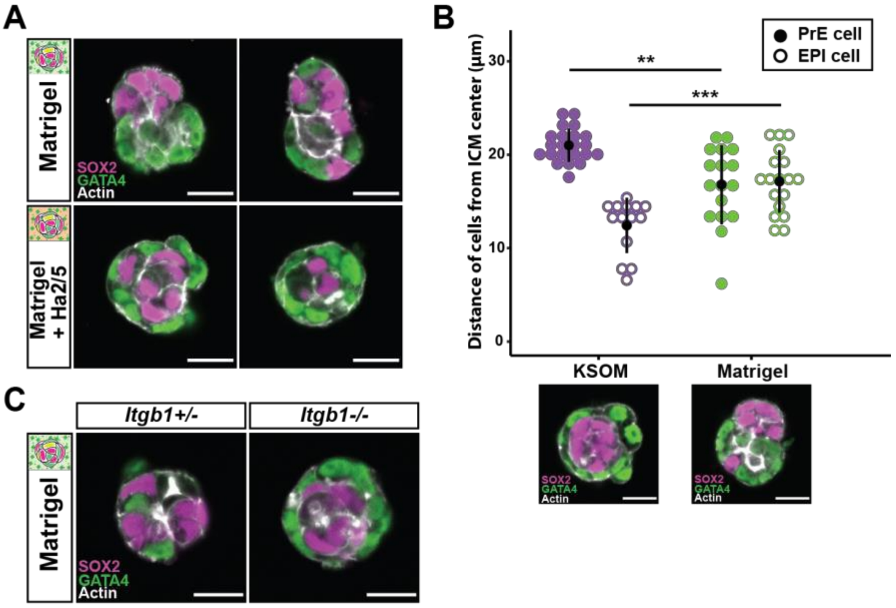

Response Figure 6. Matrigel-mediated alteration of PrE/EPI patterning requires integrin β1. (A) Additional images of images of EPI-PrE arrangement and apicobasal polarity of ICMs following culture in Matrigel or Matrigel with integrin β1-blocking antibody Ha2/5. (B) Distance of PrE and EPI cells from the center of the ICM cultured in either KSOM or Matrigel. Distance data are from representative samples displayed beneath the plot (same as images from Figure 5D and 5F). Student's *t*-test, two-sided. Error bars show mean per embryo ± s.d. n = 71 cells. $^{**}p < 0.01$, $^{***}p < 0.001$. (C) Representative images of ICMs isolated from E3.5 *Itgb1* transgenic embryos and cultured in either Matrigel. ICMs from *Itgb1+/-* embryos serve as littermate controls. SOX2 marks EPI cells, and GATA4 marks PrE cells. Scale bars = 20 µm.

Minor comments:

More info about how cell circularity was measured would be beneficial

We have added this information in the Methods section of the manuscript (highlighted).

© 2022. Published by The Company of Biologists under the terms of the Creative Commons Attribution License (https://creativecommons.org/licenses/by/4.0/).

References

Bazzoni, G., Shih, D. T., Buck, C. A. and Hemler, M. E. (1995). Monoclonal antibody 9EG7 defines a novel beta 1 integrin epitope induced by soluble ligand and manganese, but inhibited by calcium. *J. Biol. Chem.* **270**, 25570–25577.

Gonzales, M., Haan, K., Baker, S. E., Fitchmun, M., Todorov, I., Weitzman, S. and Jones, J. C. (1999). A cell signal pathway involving laminin-5, alpha3beta1 integrin, and mitogen-activated protein kinase can regulate epithelial cell proliferation. *Mol Biol Cell* **10**, 259-270.

Humphries, J. D., Schofield, N. R., Mostafavi-Pour, Z., Green, L. J., Garratt, A. N., Mould, A. P. and Humphries, M. J. (2005). Dual functionality of the anti- beta1 integrin antibody, 12G10, exemplifies agonistic signalling from the ligand binding pocket of integrin adhesion receptors. *J. Biol. Chem.* **280**, 10234–10243.

Maître, J.-L., Turlier, H., Illukkumbura, R., Eismann, B., Niwayama, R., Nédélec, F. and Hiiragi, T. (2016). Asymmetric division of contractile domains couples cell positioning and fate specification. *Nature* 1–14.

Ohnishi, Y., Huber, W., Tsumura, A., Kang, M., Xenopoulos, P., Kurimoto, K., Oleś, A. K., Araúzo-Bravo, M. J., Saitou, M., Hadjantonakis, A.-K., et al. (2014). Cell-to-cell expression variability followed by signal reinforcement progressively segregates early mouse lineages. *Nat Cell Biol* **16**, 27–37.

Wayner, E. A., Orlando, R. A. and Cheresh, D. A. (1991). Integrins alpha v beta 3 and alpha v beta 5 contribute to cell attachment to vitronectin but differentially distribute on the cell surface. *J Cell Biol* **113**, 919–929.

Wigger, M., Kisielewska, K., Filimonow, K., Plusa, B., Maleszewski, M. and Suwińska, A. (2017). Plasticity of the inner cell mass in mouse blastocyst is restricted by the activity of FGF/MAPK pathway. *Nature Publishing Group* **7**, 15136.

Second decision letter

MS ID#: DEVELOP/2021/200140

MS TITLE: ECM-integrin signalling instructs cellular position-sensing to pattern the early mouse embryo

AUTHORS: Esther Jeong Yoon Kim, Lydia Sorokin, and Takashi Hiiragi
ARTICLE TYPE: Research Article

I am happy to tell you that your manuscript has been accepted for publication in Development, pending our standard ethics checks.

Reviewer 1

Advance summary and potential significance to field

This is a high-quality paper describing the instructive roles of ECM-integrin signaling in cellular position-sensing required for tissue patterning in preimplantation mouse embryos. The authors showed that laminins and integrins are expressed at the cell-cell interface of the morula and ICM of blastocysts.
In vitro culture of the isolated inner cells in the laminin-rich ECM promoted ICM specification of the outer cells in an integrin b1-dependent manner. In Itgb1-/- embryos, PrE cells failed to polarize

© 2022. Published by The Company of Biologists under the terms of the Creative Commons Attribution License (https://creativecommons.org/licenses/by/4.0/).

and form a multilayer tissue. In vitro culture of isolated ICM in the ECM promoted outer localization of EPI cells.

This paper demonstrated that ECM-integrin signaling is a novel regulator of preimplantation development. This information will be of interest to developmental biologists especially for the scientists working on pre-implantation embryos.

Comments for the author

I am satisfied with the changes made by the authors in the revised manuscript.

Reviewer 2

Advance summary and potential significance to field

The authors have addressed by criticisms, the revised version is highly appropriate for publication in Development.

Comments for the author

No further comments.

Reviewer 3

Advance summary and potential significance to field

In their manuscript Kim and colleagues investigated the role of integrins (specifically integrin β1 activity) in lineage specification and pattern formation in pre-implantation mouse embryos. The authors in a convincing way demonstrated that that embryonic cells recognize their position within the embryo through the extracellular matrix (ECM) and integrin-mediated adhesion. Isolated early inner cells cultured in Matrigel, which is rich in laminin 111, did not form normal blastocysts but most of the cells in aggregates acquired ICM fate. Culture in Matrigel with the addition of antibody, Ha2/5 that blocks integrin β1 function resulted in restoration of wt phenotype. A similar effect was achieved via inhibition of integrin α6 activity. Similarly, upon genetic ablation of Itgb1, integrin β1-deficient cells were refractory to the effects of Matrigel. At the same time PrE cells failed to resolve into a single monolayer in Itgb1-/- embryos.

Comments for the author

In their revised manuscript, Hiiragi and colleagues have done most of the changes suggested by this reviewer and I am fully satisfied with their response to my comments. This beautiful work fully deserved to be presented to the developmental biologist community

© 2022. Published by The Company of Biologists under the terms of the Creative Commons Attribution License (https://creativecommons.org/licenses/by/4.0/).
